# Supplementary material for: Dynamic BH3 profiling identifies pro-apoptotic drug combinations for the treatment of malignant pleural mesothelioma
Source: Nat Commun. 2023 May 20;14:2897. doi: 10.1038/s41467-023-38552-z (PMC10199949; doi:10.1038/s41467-023-38552-z)
Supplement: Supplementary file 1 — Supplementary information [file 41467_2023_38552_MOESM1_ESM.pdf]

Supplementary Information

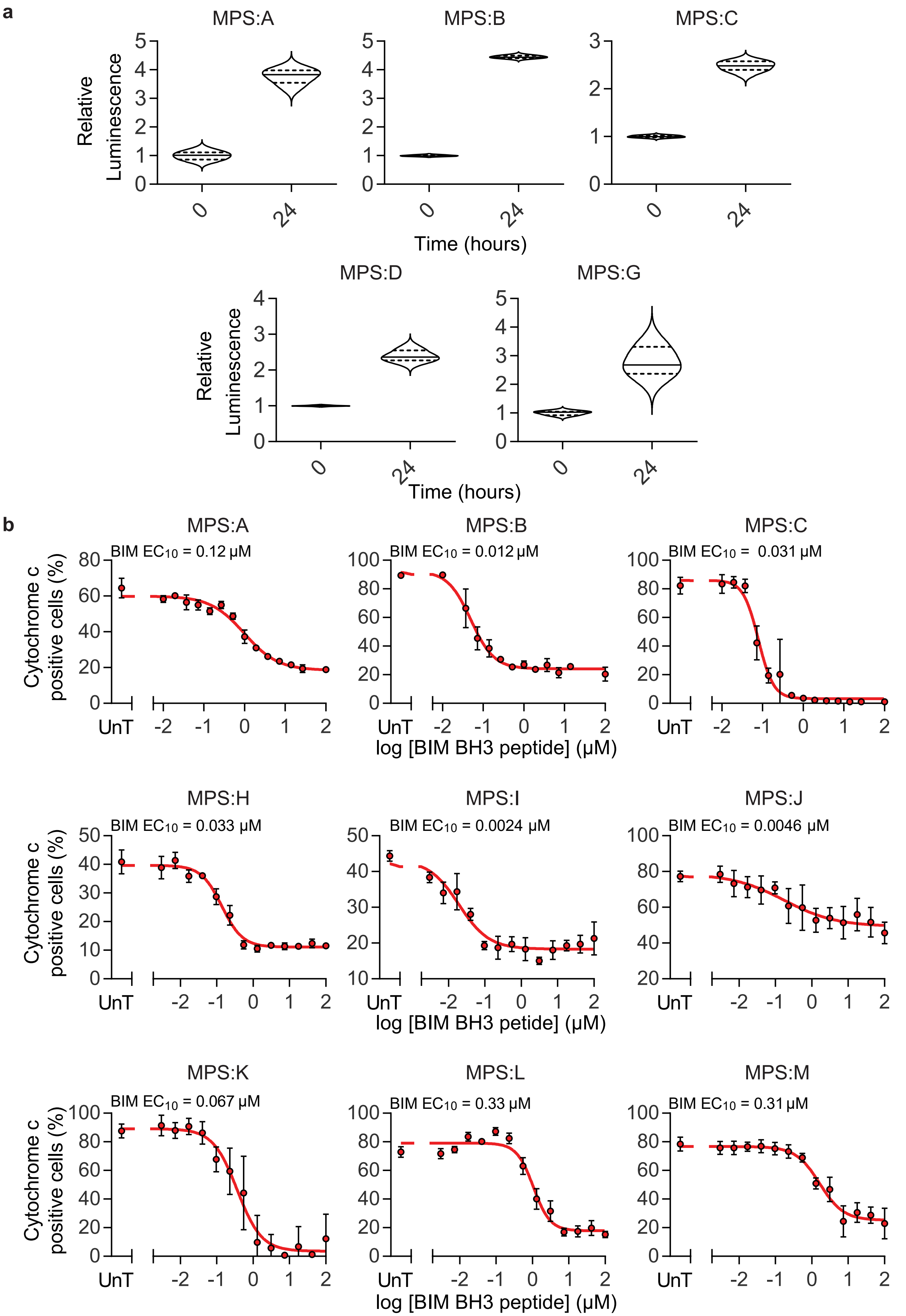

Supplementary Figure 1: Primary MPM cells over 24 hours ex vivo and BIM BH3 peptide dose response to calculate optimum BIM concentration for HTDBP. (**a**) Primary MPM cells were seeded in a 384-well plate and Cell Titer Glo carried out according to manufacturer's instructions at 0 and 24 hours post seeding. Data presented as violin plots with median as solid line and quartiles as dashed line. Carried out in technical replicates, MPS:A n=6, MPS:B n=4, MPS:C n=10, MPS:D n=4 and MPS:G n=3.(**b**) Graph showing BIM BH3 peptide dose response with error bars (mean ± standard deviation) on untreated primary MPM tumor cells. To calculate the optimum BIM BH3 peptide concentration (EC_{10}) for HTDBP for that specific patient sample. Carried out in technical replicates, MPS:A n=4, MPS:B n=3, MPS:C n=8, MPS:H n=6, MPS:I n=3, MPS:J n=9, MPS:K n=6, MPS:L n=6 and MPS:M n=6.

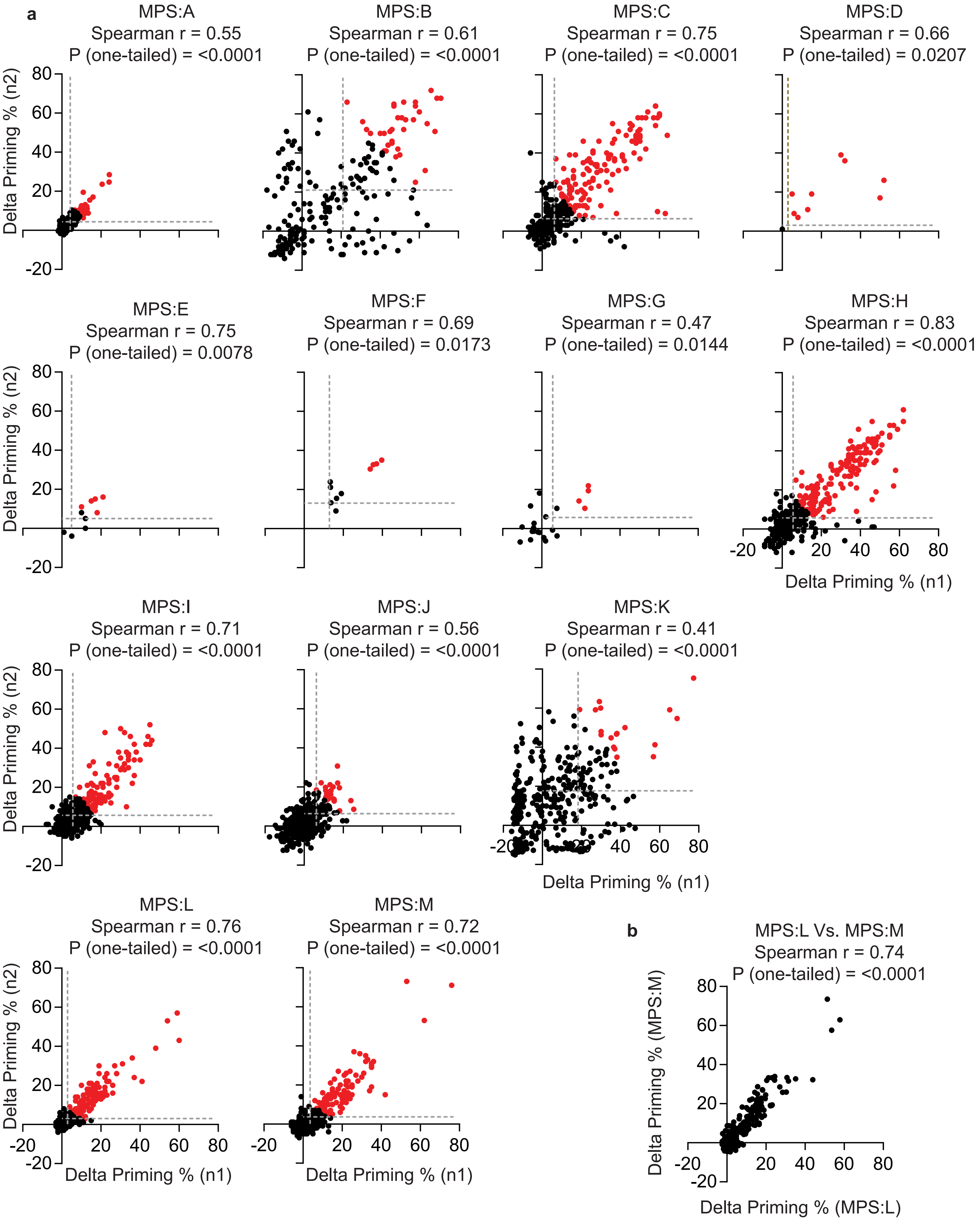

Supplementary Figure 2: Correlation of CROCS HTDBP replicates in primary MPM patient samples. Primary MPM cells were treated as previously described in Figure 1. (**a**) Graphs show delta priming % (measure of drug-induced priming compared to DMSO-control for each drug treatment) correlation between replicates for individual drug treatments, using one-tailed Spearman ranked test, for each MPM patient samples mentioned in Figure 1. P values are stated on each graph for each individual patient sample. Red dot indicates a hit in the assay (Z-score ≥ 3 with no replicate < 1.5). Grey dashed line indicates Z-score 1.5 for that sample. (**b**) Graph showing the mean delta priming % correlation between two tumor samples from the same patient (MPS:L and MPS:M) using one-tailed Spearman ranked test (P value = <0.0001). MPS:L is a tumor from the 7th rib and MPS:M is a pleural tumor.

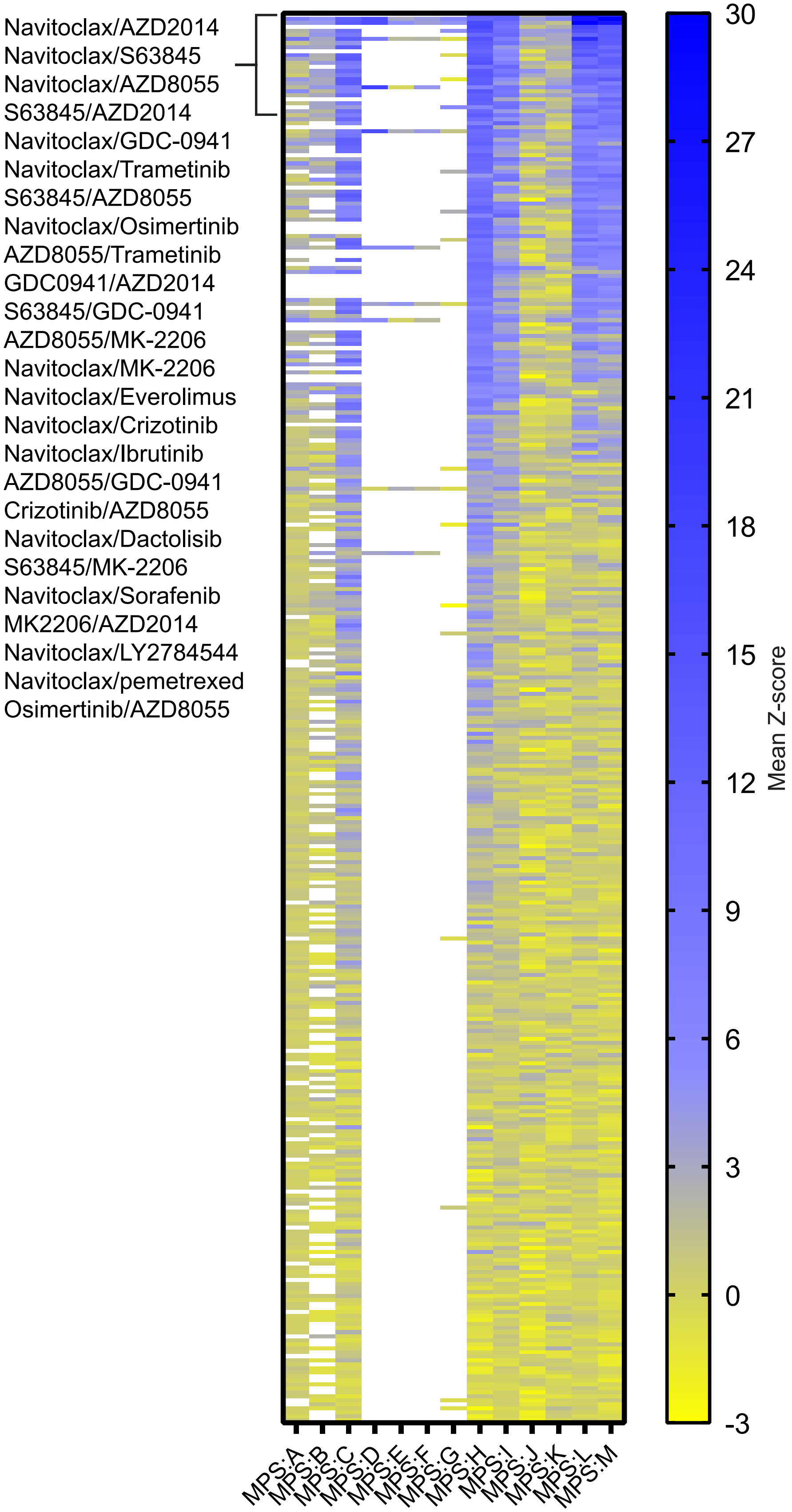

Supplementary Figure 3: Heatmap of CROCS HTDBP hits on primary MPM patient samples. Fresh primary MPM patient samples were dissociated, treated with CROCS and HTDBP carried out as described in Figure 1. Cells are analyzed by immunofluorescence microscopy and Z-score calculated to identify drug/drug combinations that prime tumor cells. Heatmap showing ranked (highest at the top) mean Z-score for all MPM patient samples (MPS) for each drug treatment. Blue represents a hit with a Z-score ≥ 3 with no replicate <1.5. Yellow are non-hits.

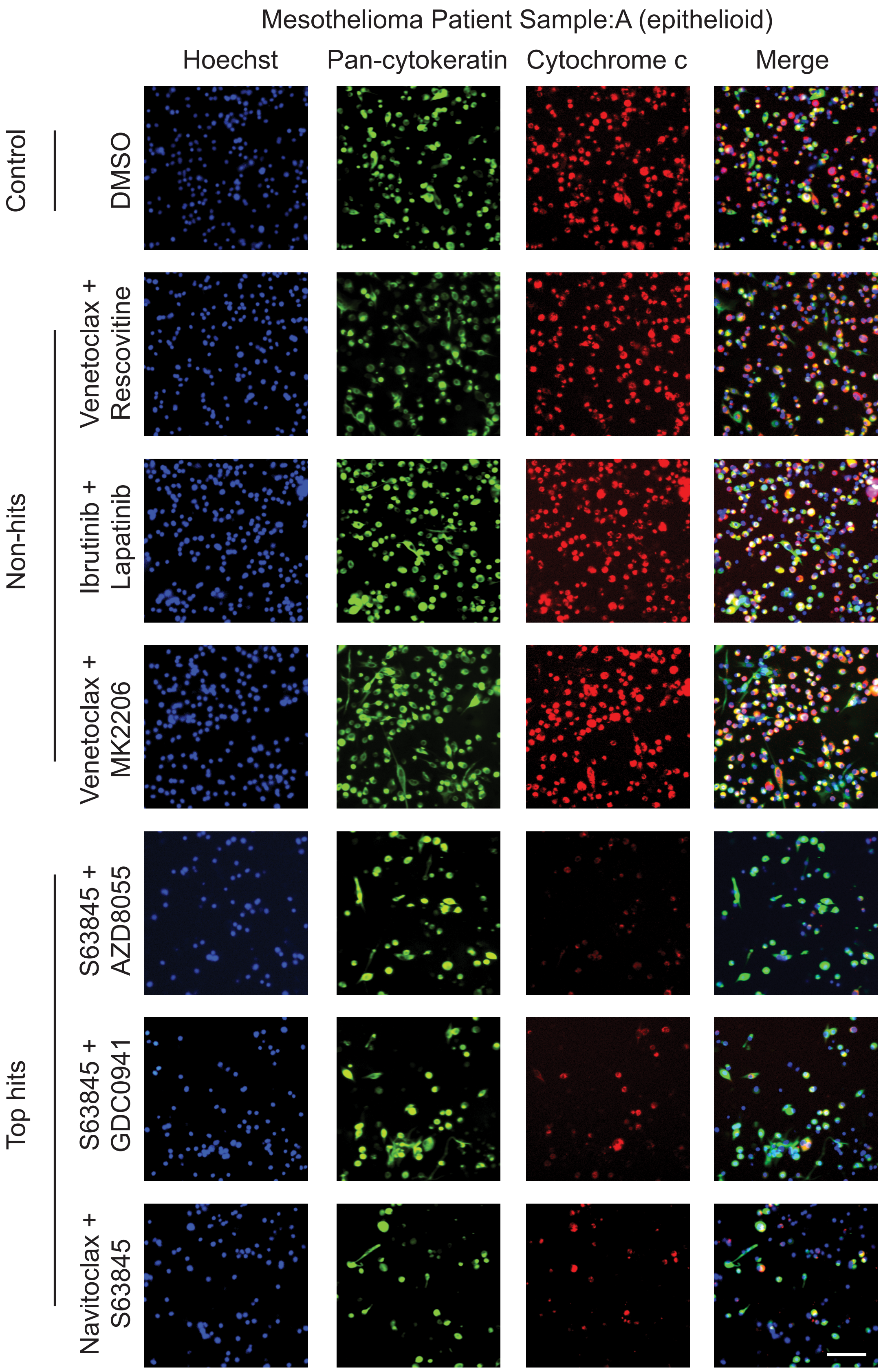

Supplementary Figure 4: Representative CROCS HTDBP microscopy images of epithelioid primary MPM cells. Epithelioid primary MPM cells were treated as previously described in Figure 1, in technical duplicate. Representative immunofluorescence microscopy images from epithelioid MPM patient sample A (MPS:A). Images taken at 10-fold magnification. Hoechst 33342 stains DNA/nucleus (blue) to identify the number of cells per each well. Pan-cytokeratin-488 antibody (green) identifies tumor cells (parent population). From the parent population the cytochrome c positive cells % (red) was calculated. DMSO treatment is a negative control for cytochrome c loss. Non-hits are drug treatments that didn't score a Z-score ≥ 3 (no drug-induced priming). Top hits are the top 3 drug treatments (highest drug-induced priming/largest Z-score) for this patient sample and first 3 red dots in Figure 1B MPS:A. Scale bar is 100 um.

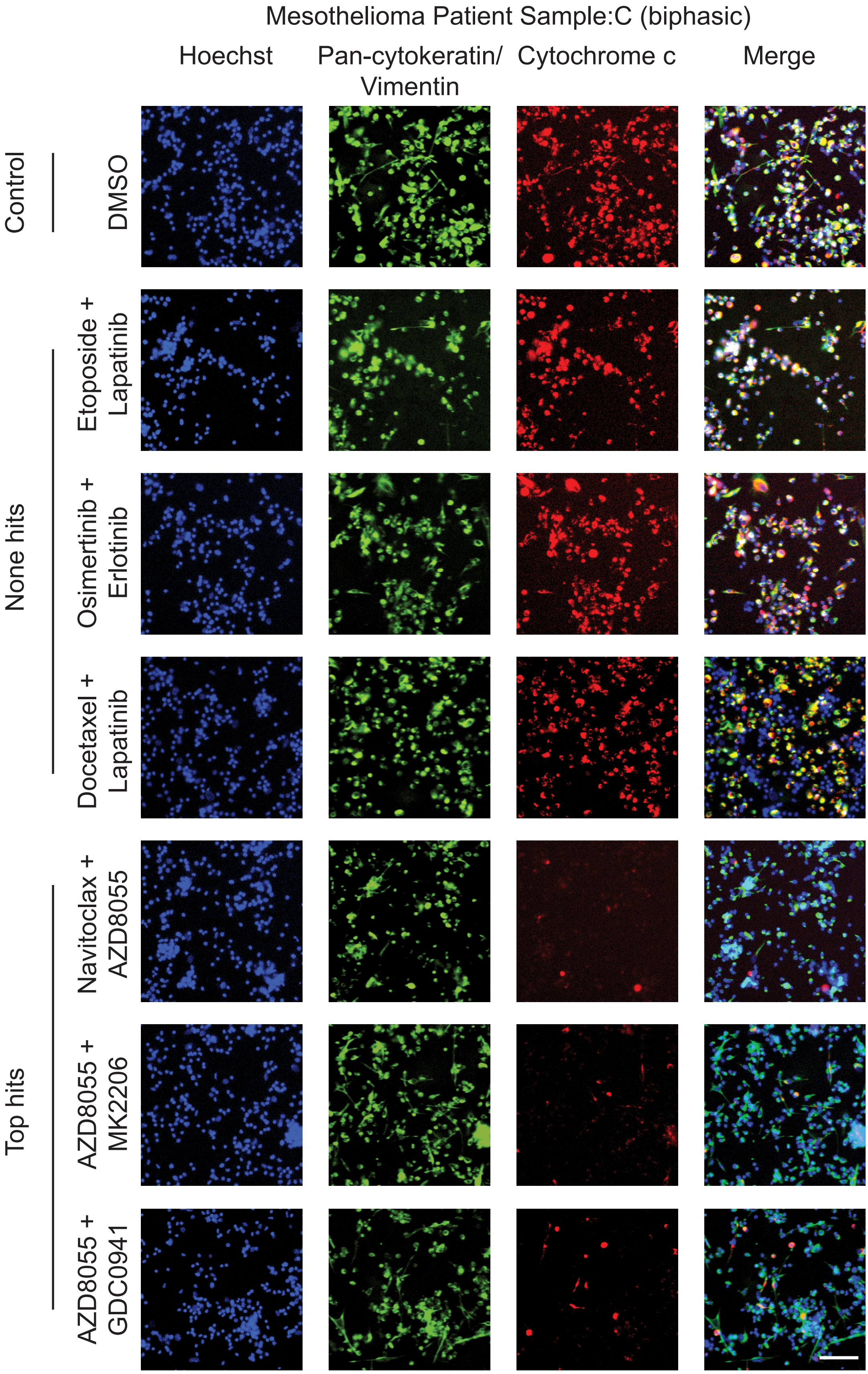

Supplementary Figure 5: Representative CROCS HTDBP microscopy images of biphasic primary MPM cells. Biphasic primary MPM cells were treated as previously described in Figure 1, in technical duplicate. Representative immunofluorescence microscopy images from biphasic MPM patient sample C (MPS:C). Images taken at 10-fold magnification. Hoechst 33342 stains DNA/nucleus (blue) to identify the number of cells per each well. Pan-cytokeratin-488/vimentin-488 antibody (green) identifies tumor cells (parent population). From the parent population the cytochrome c positive cells % (red) was calculated. DMSO treatment is a negative control for cytochrome c loss. Non-hits are drug treatments that didn't score a Z-score ≥ 3 (no drug-induced priming). Top hits are the top 3 drug treatments (highest drug-induced priming/largest Z-score) for this patient sample and first 3 red dots in Figure 1B MPS:C. Scale bar is 100 um.

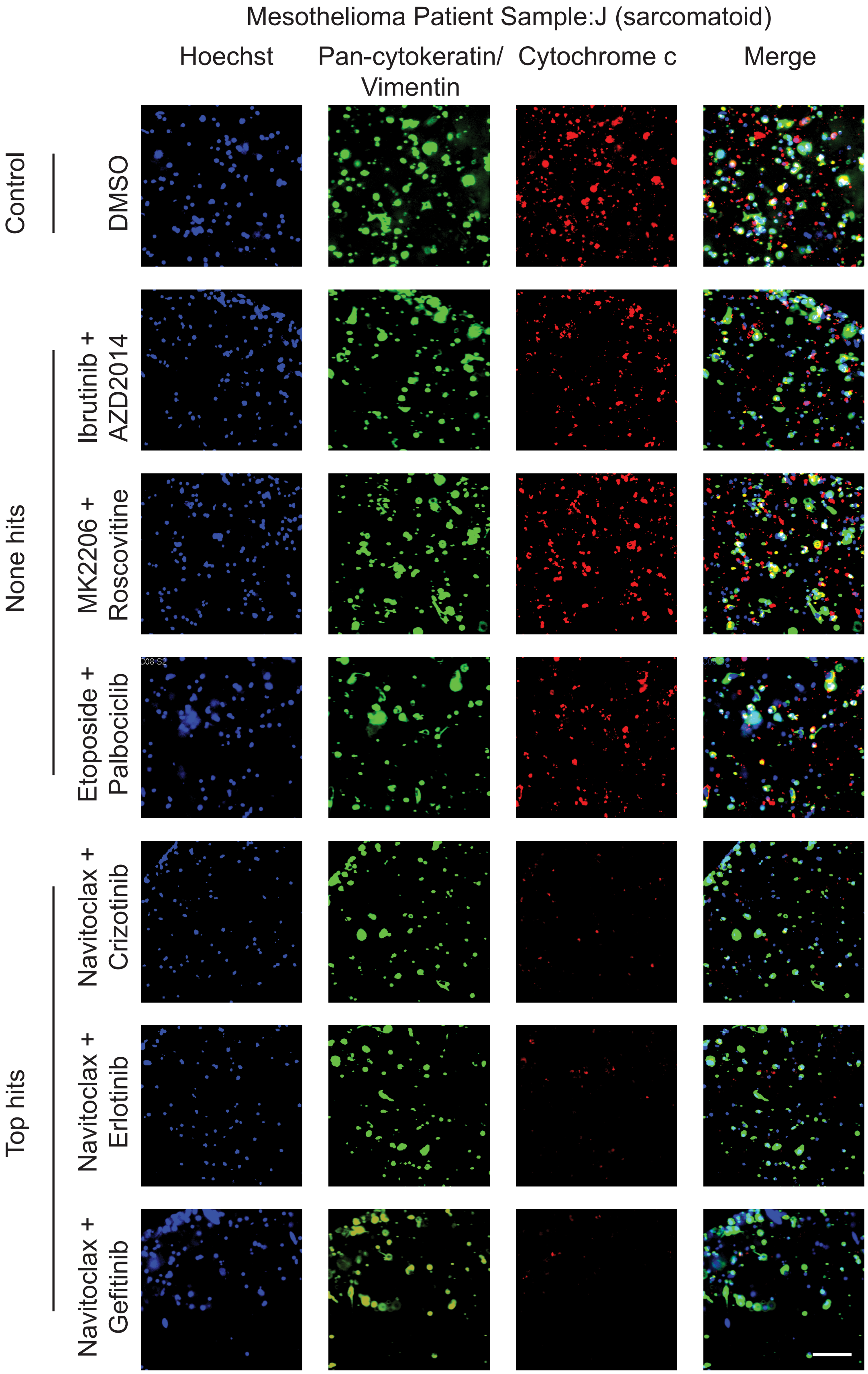

Supplementary Figure 6: Representative CROCS HTDBP microscopy images of sarcomatoid primary MPM cells. Sarcomatoid primary MPM cells were treated as previously described in Figure 1, in technical duplicate. Representative immunofluorescence microscopy images from sarcomatoid MPM patient sample J (MPS:J). Images taken at 10-fold magnification. Hoechst 33342 stains DNA/nucleus (blue) to identify the number of cells per each well. Pan-cytokeratin-488/vimentin-488 antibody (green) identifies tumor cells (parent population). From the parent population the cytochrome c positive cells % (red) was calculated. DMSO treatment is a negative control for cytochrome c loss. Non-hits are drug treatments that didn't score a Z-score ≥ 3 (no drug-induced priming). Top hits are the top 3 drug treatments (highest drug-induced priming/largest Z-score) for this patient sample and first 3 red dots in Fig. 1B MPS:J. Scale bar is 100 um.

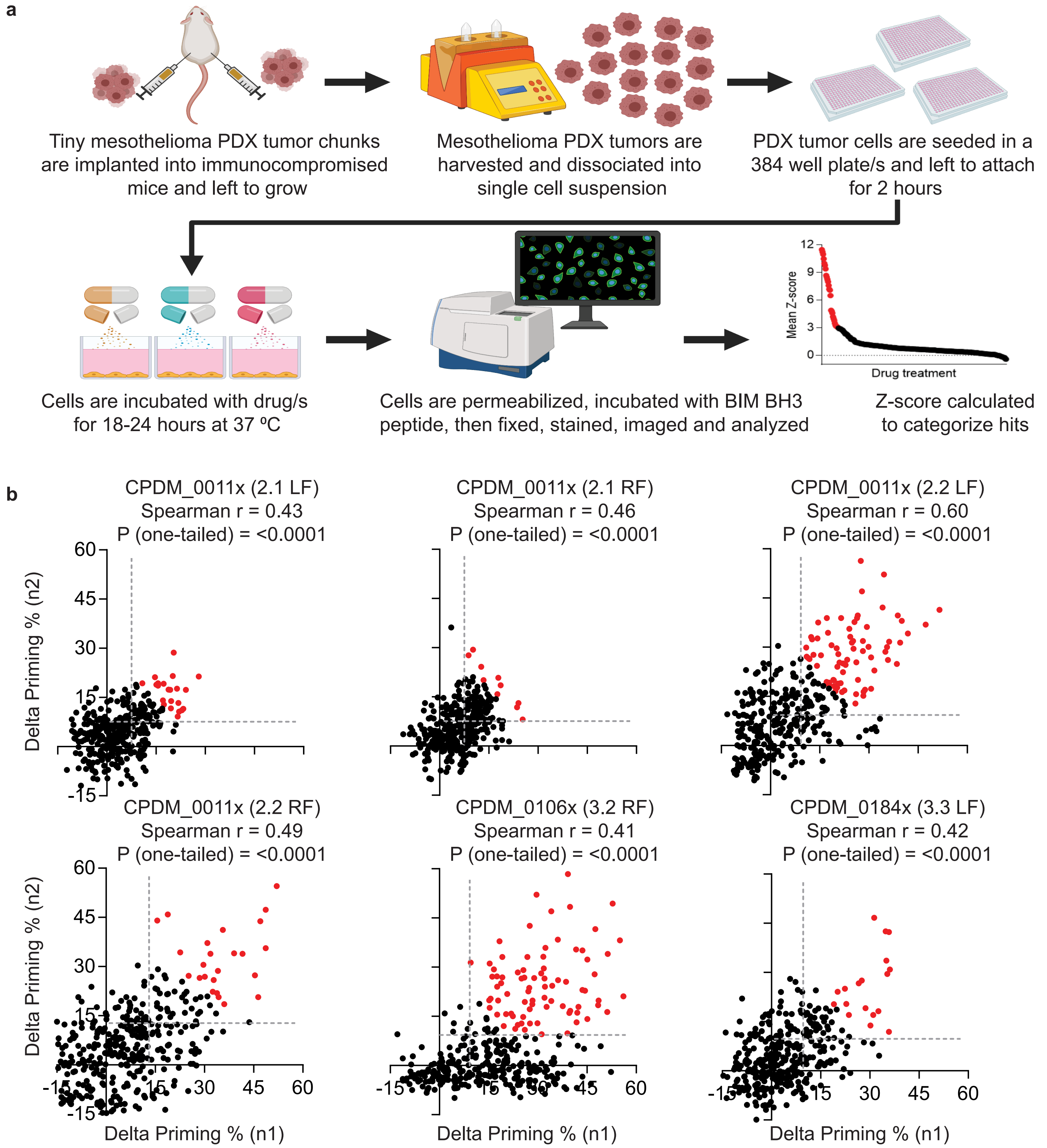

Supplementary Figure 7: Clinically relevant oncology combination screen (CROCS) on MPM PDX models using HTDBP to identify hits. Malignant pleural mesothelioma PDX's were dissociated when they reached ~1000 mm3 and treated with CROCS and HTDBP carried out. Cells analyzed by immunofluorescence microscopy and Z-score calculated to identify drug/drug combinations that prime PDX tumor cells. (**a**) Schematic showing the workflow for measuring drug-induced priming using CROCS HTDBP on MPM PDX samples. Created with BioRender.com. (**b**) Graphs show delta priming % (measure of drug-induced priming compared to DMSO-control for each drug treatment) correlation between replicates, using one-tailed Spearman ranked test, for individual drug treatments in MPM PDX tumor harvested in Fig. 3A. P values are shown on each graph for each PDX model. Model CPDM_0011x had 4 tumors harvested, CPDM_0106x and CPDM_0184x had 1 tumor each harvested. Each individual dot on the correlation graph represents a different drug treatment (single agent or drug-drug combination). A red dot represents a hit with a Z-score ≥ 3 with no replicate <1.5. Black dots are non-hits.

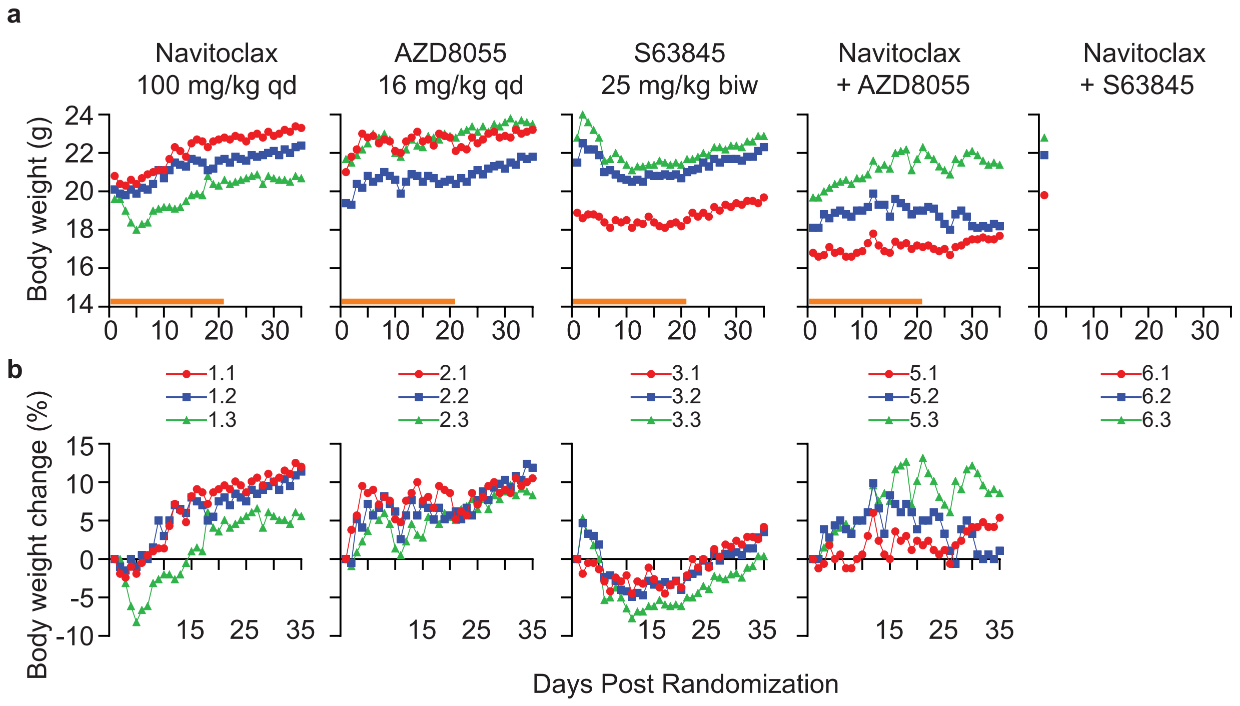

Supplementary Figure 8: In vivo tolerance study with top ex vivo HTDBP hits. A tolerance study was carried out on non-tumor bearing SCID-beige mice (host mice strain) to assess if the top hit drug combinations identified from CROCS HTDBP in MPM primary and PDX samples was tolerable in mice. Combinations carried out with well-known efficacious and tolerable single agent doses of navitoclax, AZD8055 and S63845 indicated in the Figure. Three mice per treatment group. (**a**) Graph shows individual mouse body weight of three mice over the 21 days of dosing (indicated by orange line) and two weeks after dosing had finished. (**b**) Graph shows body weight/change for three individual mice (based on day 0 body weight) over time.

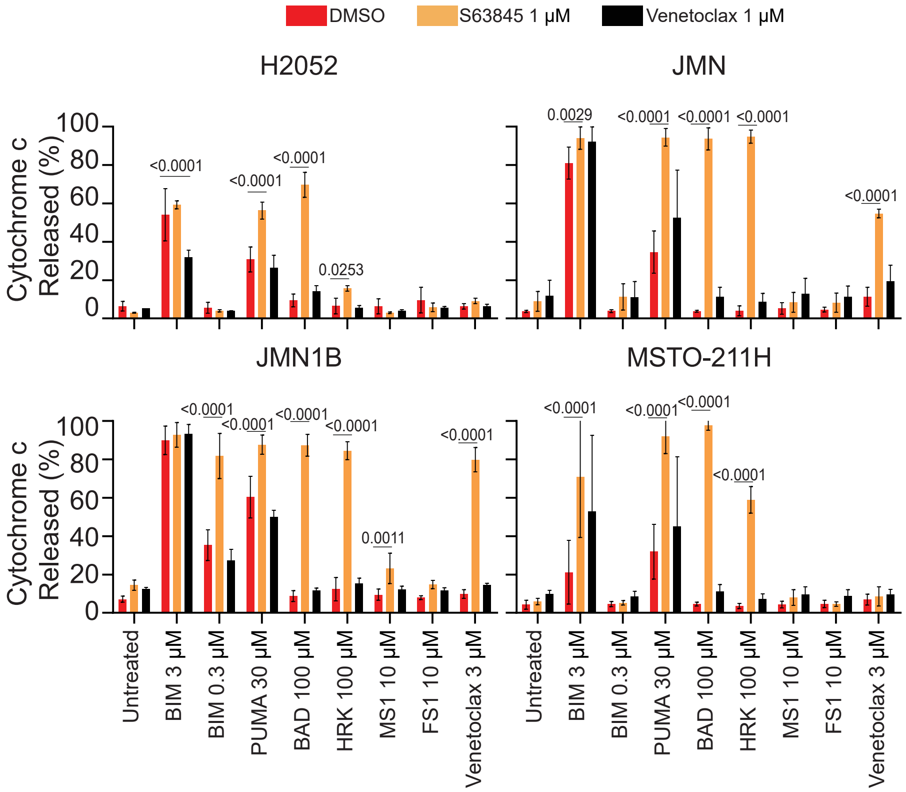

Supplementary Figure 9: The molecular mechanism of navitoclax plus AZD8055 combination in vitro in MPM. Analysis of drug-induced priming and anti-apoptotic dependencies after treatment with S63845 and venetoclax in MPM cell lines (H2052, JMN, JMN1B and MSTO-211H). Overall priming is measured by BIM or PUMA, whereas HRK, MS1, FSI and venetoclax are specific for BCL-xL, MCL-1, BFL-1 and BCL-2 dependency respectively. Cells treated with 1 μM S63845 or venetoclax and then DBP carried out. Cytochrome c positive cells % (cytochrome c released = 100 - cytochrome c positive cells %) was measured using immunofluorescence microscopy, on permeabilized cells after 1 hour incubation with indicated BH3 peptide concentration. Data are presented in bar graphs as mean values (n=4 independent experiments for DMSO and n=3 independent experiments for S63845 and venetoclax treatment) with error bars (± standard deviation). We calculated significance using a 2-way ANOVA multiple comparisons test to DMSO-control (n=3). Significance was only observed when comparing DMSO Vs. S63845 treatment (not with DMSO Vs. venetoclax treatment) and therefore the p values shown on the graph are comparing DMSO Vs. S63845 for indicated BH3 peptide.

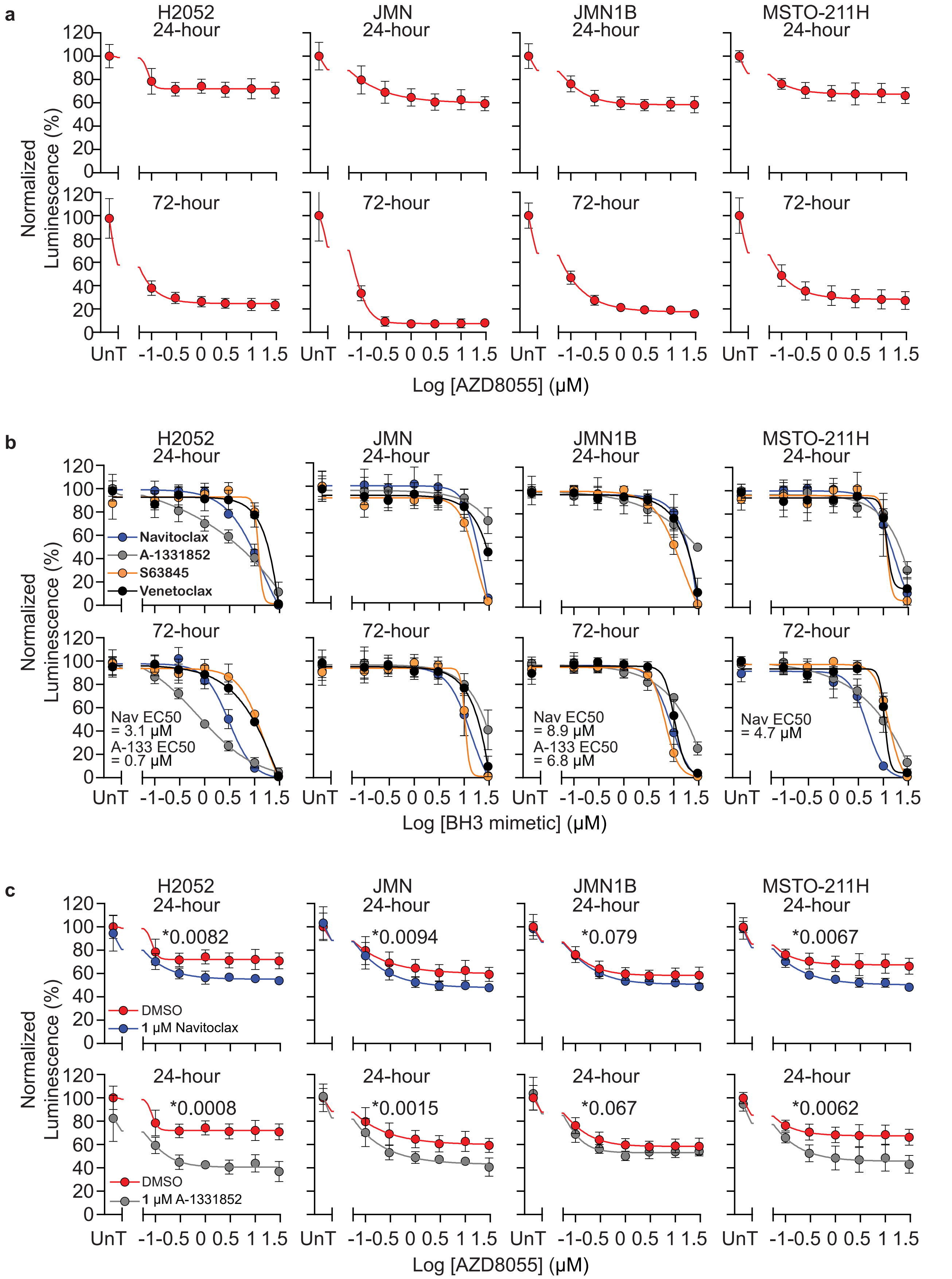

Supplementary Figure 10: AZD8055 and BH3 mimetic dose response curves in MPM cell lines. Malignant pleural mesothelioma cell lines (H2052, JMN, JMN1B and MSTO-211H) were exposed with the indicated concentration of (**a**) AZD8055 (n=3 independent experiments) or (**b**) BH3 mimetics navitoclax, A-1331852, S63845 or venetoclax for 24 or 72 hours (n=3 independent experiments). (**c**) Cell lines treated with AZD8055 as in part (a) in combination with either DMSO-control (AZD8055-only), 1 µM navitoclax or A-1331852. * significance was based on one-tailed unpaired t-test versus DMSO-treated AZD8055 area under curve (AUC) for the same cell line. Cell viability was assessed using Cell Titer Glo. Graph represents dose response curve with error bars (mean ± standard deviation) for n=3 independent experiments.

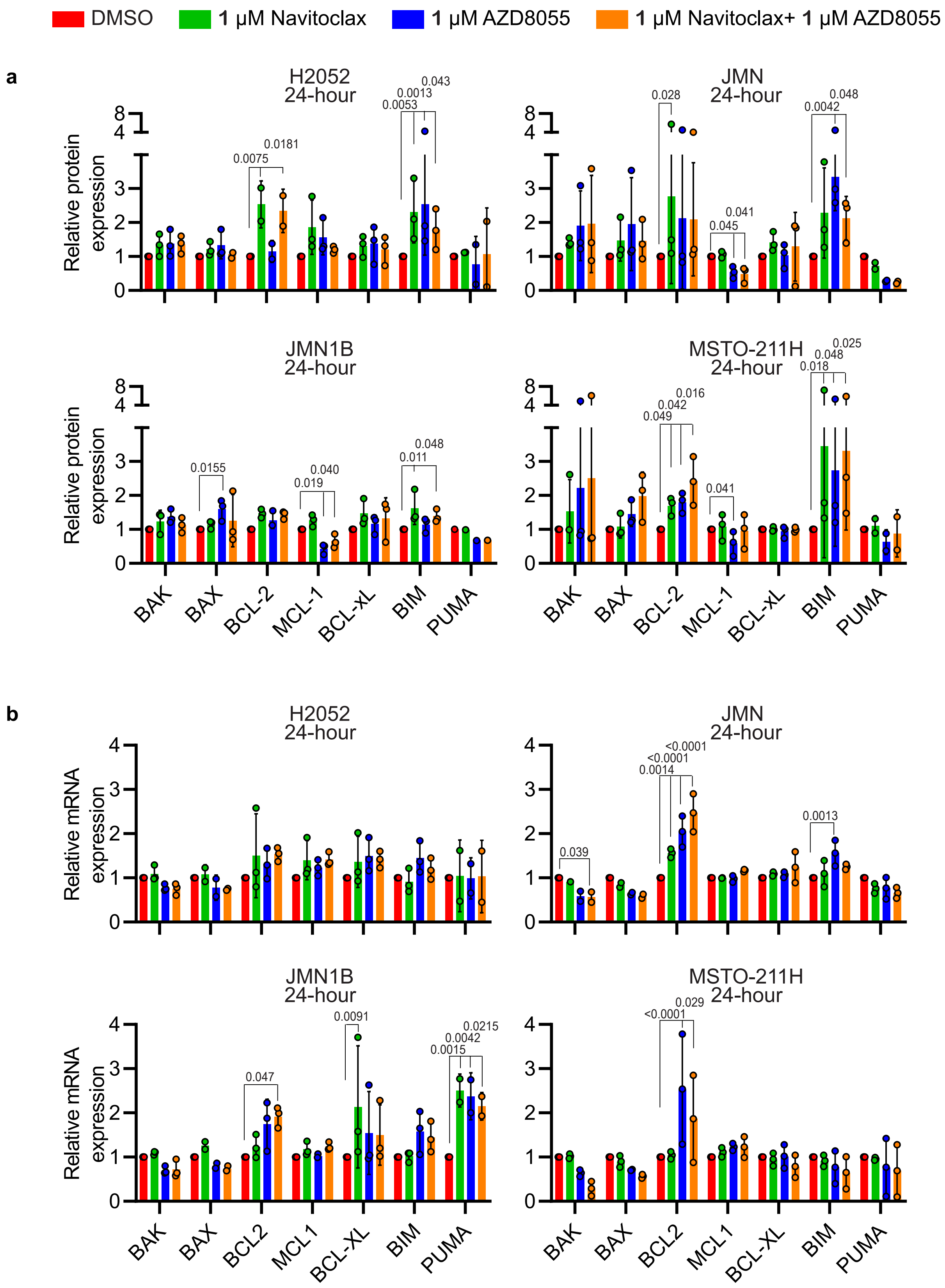

Supplementary Figure 11: Quantification of protein and mRNA levels for BCL-2 family members after treatment with navitoclax, AZD8055 or navitoclax plus AZD8055 combination in MPM cell lines. MPM cell lines (H2052, JMN, JMN1B and MSTO-211H) treated with the indicated concentration of navitoclax, AZD8055 or navitoclax plus AZD8055 combination for 24 hours. Data presented as bar graph as mean values showing corresponding data points for n=3 quantification of proteins levels by Immunoblots (**a**) or n=3 mRNA levels by qPCR (**b**) in MPM cell line for, BAK, BAX, BCL-2, MCL-1, BCL-xL, BIM and PUMA. BCL-2 family members were normalized to loading control (immunoblots) or house keeping gene (qPCR) and then shown relative to DMSO-control levels for that specific protein (a) or gene (b). Significance was calculated using a 2-way ANOVA multiple comparisons test to DMSO-control, p values are shown on graph.

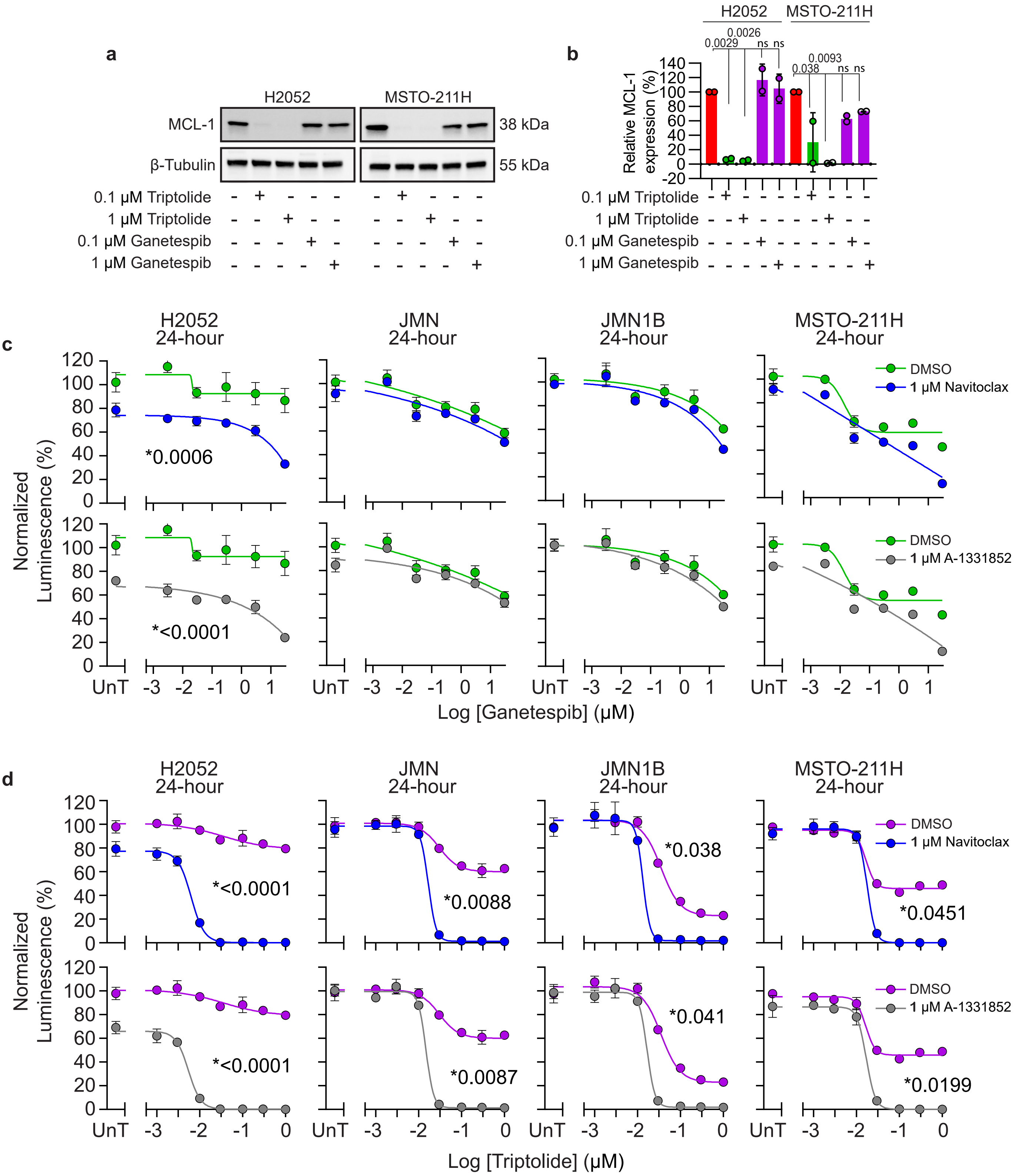

Supplementary Figure 12: Ganetespib or triptolide in combination with BCL-xL antagonist in MPM cell lines. Malignant pleural mesothelioma cell lines (H2052, JMN, JMN1B and MSTO-211H) were exposed with the indicated concentration ganetespib or triptolide for 24 hours. (**a**) Representative immunoblots of MPM cell lysates after 24 hours treatment with indicated concentration of drug for, MCL-1 and β-Tubulin. N= 2 independent experiments. (**b**) Data presented as bar graph as mean values showing corresponding data points for n=2 quantification of two independent immunoblots for MCL-1 protein levels relative to DMSO-control for part (a). Significance was based on one-way ANOVA multiple comparisons test, comparing to DMSO-control. (**a-b**) Cell lines treated with a dose response to ganetespib (**c**) or triptolide (**d**) for 24 hours in combination with either DMSO-control (ganetespib-only or triptolide-only), **1** µM Navitoclax or **1** µM A-1331852. * significance was based on one-tailed unpaired t-test versus ganetespib-only or triptolide-only area under curve (AUC) for the same cell line. P values are shown on graph. Cell viability was assessed using Cell Titer Glo. Graph represents dose response curve with error bars (mean ± standard deviation) for n=3 independent experiments.

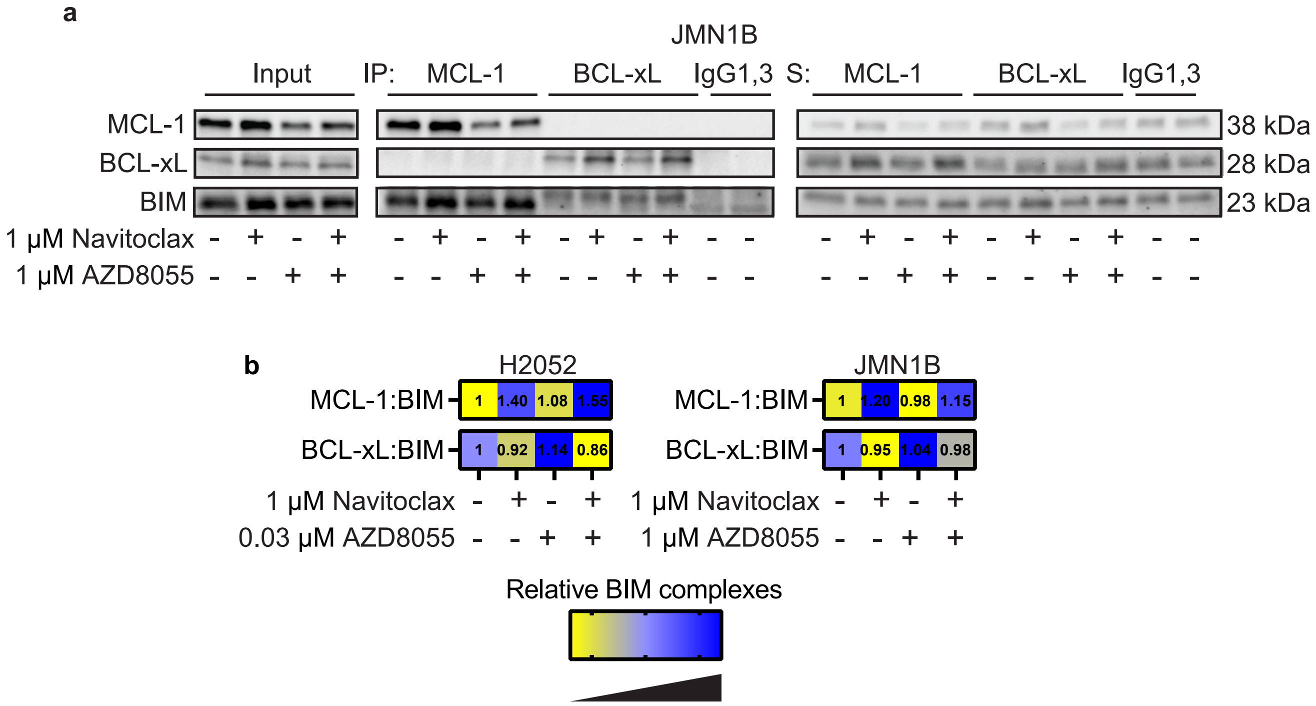

Supplementary Figure 13: BIM complexes with BCL-xL or MCL-1 after treatment with navitoclax, AZD8055 or navitoclax plus AZD8055 combination in MPM cells. (**a**) 24 hours after treatment with indicated drug concentration, BCL-xL and MCL-1 were immunoprecipitated in JMN1B cells and BIM complexes were determined by Western blotting analysis (Input total cell lysate; IP, immunoprecipitated fraction; IgG1 (immunoglobulin isotype 1; MCL-1 isotype) and IgG3 (immunoglobulin isotype 3; BCL-xL isotype) control; S, supernatant). N=2 independent experiments. (**b**) Heat map of densitometry analysis carried out on BIM immunoblot for MCL-1 or BCL-xL IP for H2052 cells (Figure 5e) or JMN1B cells (part (a) of this Figure). BIM complexes to either MCL-1 or BCL-xL after 24 hours drug treatment with indicated drug concentration was calculated relative to DMSO-control. Relative values included in the heat map.

Table S1: Drugs included in CROCS, mechanism of action and clinical status

Drug name	Drug target	Drug class	Trials/clinic	Source
Navitoclax (ABT-263)	BCL-2/BCL-xL/BCL-w	BH3 mimetic	Phase 3	Medchem express
S63845	MCL-1	BH3 mimetic	Phase 1	Medchem express
Venetoclax (ABT199)	BCL-2	BH3 mimetic	Clinic	Medchem express
Etoposide	Topoisomerase 2 inhibitor	Chemotherapy	Clinic	Sigma Aldrich
Docetaxel (Taxotere)	Blocks mitotic spindle depolymerisation	Chemotherapy	Clinic	Selleck
Paclitaxel (Taxol)	Blocks mitotic spindle depolymerisation	Chemotherapy	Clinic	Selleck
Pemetrexed	inhibits DNA and RNA synthesis	Chemotherapy	Clinic	Selleck
Osimertinib	EGFR T790M L858R	Tyrosine kinase inhibitor	Clinic	Selleck
AZD8055	mTOR inhibitor (mTORC1/2)	Small molecule inhibitor	Phase 1	Medchem express
AZD2014	mTOR inhibitor (mTORC1/2)	Small molecule inhibitor	Phase 2	Selleck
Everolimus	mTOR inhibitor (mTORC1)	Small molecule inhibitor	Clinic	Medchem express
GDC0941 (Pictilisib)	Pan PI3K inhibitor	Small molecule inhibitor	Phase 2	Fisher Scientific
Dactolisib (BEZ235)	Dual PI3K/mTOR inhibitor	Small molecule inhibitor	Phase 2	Medchem express
MK2206	Pan AKT inhibitor	Small molecule inhibitor	Phase 2	Selleck
Crizotinib	ALK	Tyrosine kinase inhibitor	Clinic	Selleck
Erlotinib	EGFR	Tyrosine kinase inhibitor	Clinic	Haoyuan chemexpress
Gefitinib	EGFR	Tyrosine kinase inhibitor	Clinic	Selleck
Ibrutinib (PCI-32765)	Tec kinase inhibitor	Small molecule inhibitor	Clinic	ApexBio
Lapatinib	Dual Her2/EGFR inhibitor	Tyrosine kinase inhibitor	Clinic	Medchem express
Palbociclib	CDK4/CDK6 inhibitor	Small molecule inhibitor	Clinic	Medchem express
Regorafenib	VEGFR2-TIE2 inhibitor	Tyrosine kinase inhibitor	Clinic	Haoyuan chemexpress
Seliciclib (Roscovitine)	Cdc2, CDK2, CDK5 inhibitor	Small molecule inhibitor	Phase 2	Haoyuan chemexpress
Sorafenib	Raf-1, B-Raf, VEGFR-2	Tyrosine kinase inhibitor	Clinic	LC Laboratories
Sunitinib	VEGFR2, PDGFRβ, c-Kit	Tyrosine kinase inhibitor	Clinic	Medchem express
Trametinib	MEK1/2 inhibitor	Small molecule	Clinic	Medchem express
LY2784544	JAK1/2/3	Small molecule inhibitor	Phase 2	Medchem express

Table S2: Malignant pleural mesothelioma patient sample cellularity

MPM Patient Sample ID	Size mg	Number of cells	Cells/mg
MPS:A	17,000	70,000,000	4,118
MPS:B	856	2,700,000	3,154
MPS:C	5,300	50,000,000	9,434
MPS:D	437	325,000	744
MPS:E	387	170,000	439
MPS:F	355	175,000	493
MPS:G	1,013	310,000	306
MPS:H	829	1,220,000	1,472
MPS:I	2,770	80,500,000	29,061
MPS:J	3,829	6,900,000	1,802
MPS:K	1,234	25,875,000	20,968
MPS:L	701	13,350,000	19,044
MPS:M	930	10,125,000	10,887

Table S3: Drugs included in CROCS for each MPM patient sample

	MPS:A	MPS:B	MPS:C	MPS:D	MPS:E	MPS:F	MPS:G	MPS:H	MPS:I	MPS:J	MPS:K	MPS:L	MPS:M	Total
Navitoclax (ABT-263)	✓	✓	✓	✓	✓	✓	✓	✓	✓	✓	✓	✓	✓	13
S63845	✓	✓	✓	✓	✓	✓	✓	✓	✓	✓	✓	✓	✓	13
AZD8055	✓	✓	✓	✓	✓	✓	✓	✓	✓	✓	✓	✓	✓	13
Dactolisib (BEZ235)	✓	✓	✓	✓	✓	✓		✓	✓	✓	✓	✓	✓	12
Etoposide	✓	✓	✓				✓	✓	✓	✓	✓	✓	✓	10
GDC-0941 (Pictilisib)	✓	✓	✓				✓	✓	✓	✓	✓	✓	✓	10
Docetaxel (Taxotere)	✓	✓	✓					✓	✓	✓	✓	✓	✓	9
Paclitaxel (Taxol)	✓	✓	✓					✓	✓	✓	✓	✓	✓	9
Osimertinib	✓	✓	✓					✓	✓	✓	✓	✓	✓	9
Venetoclax (ABT199)	✓	✓	✓					✓	✓	✓	✓	✓	✓	9
Crizotinib	✓	✓	✓					✓	✓	✓	✓	✓	✓	9
Erlotinib	✓	✓	✓					✓	✓	✓	✓	✓	✓	9
Ibrutinib (PCI-32765)	✓	✓	✓					✓	✓	✓	✓	✓	✓	9
Lapatinib	✓	✓	✓					✓	✓	✓	✓	✓	✓	9
MK2206	✓	✓	✓					✓	✓	✓	✓	✓	✓	9
Sorafenib	✓	✓	✓					✓	✓	✓	✓	✓	✓	9
Sunitinib	✓	✓	✓					✓	✓	✓	✓	✓	✓	9
Trametinib	✓	✓	✓					✓	✓	✓	✓	✓	✓	9
LY2784544	✓	✓	✓					✓	✓	✓	✓	✓	✓	9
Pemetrexed		✓	✓				✓	✓	✓	✓	✓	✓	✓	9
Gefitinib	✓		✓					✓	✓	✓	✓	✓	✓	8
Everolimus	✓		✓					✓	✓	✓	✓	✓	✓	8
Palbociclib	✓		✓					✓	✓	✓	✓	✓	✓	8
Regorafenib	✓		✓					✓	✓	✓	✓	✓	✓	8
Seliciclib (Roscovitine)	✓		✓					✓	✓	✓	✓	✓	✓	8
AZD2014								✓	✓	✓	✓	✓	✓	6

Table S4, Top hits in Epithelioid Vs. Bhiphasic patient samples

	**MPM ID:**	**MPS:A**	**MPS:B**	**MPS:D**	**MPS:K**	**MPS:L**	**MPS:M**	**MPS:C**	**MPS:E**	**MPS:F**	**MPS:G**	**MPS:H**	**MPS:I**	**MPS:J**	
	**Histology:**	E	E	E	E	E	E	B	B	B	B	B	B	S	
	**BIM EC_{10} (μM)**	0.12	0.012	N/A	0.067	0.33	0.31	0.031	N/A	N/A	N/A	0.033	0.0024	0.0046	
	**Drug treatments**	Z-score	Z-score	Z-score	Z-score	Z-score	Z-score	Z-score	Z-score	Z-score	Z-score	Z-score	Z-score	Z-score	Total
1	Navitoclax/S63845	7.9	4.9	16.0	6.3	25.7	29.9	13.0	3.2	4.0	6.5	11.9	12.9	3.8	13
2	Navitoclax/AZD8055	4.0	4.4	16.1		28.9	25.6	14.1	4.9	3.8	4.6	16.6	10.8		11
3	S63845/AZD8055	9.4	3.2	8.0	3.2	22.0	13.1	13.2				15.3	7.8		9
4	Navitoclax/GDC-0941		4.0		4.1	15.8	12.9	10.5			4.8	15.6	10.5	3.7	9
5	Navitoclax/Dactolisib		4.3	18.5	5.2	7.1	10.5	7.0		4.4			9.4	3.6	9
6	Navitoclax		3.3	16.4		6.4	7.0	8.3		4.2		10.4	8.0	4.2	9
7	Navitoclax/Trametinib		4.4		3.2	12.2	13.8	8.3				11.2	12.0	3.8	8
8	AZD8055/GDC-0941	4.3	3.0		3.2	11.5	13.6	13.8				13.0	5.2		8
9	Crizotinib/AZD8055	3.1	3.1		3.5	11.1	7.7	13.3				13.2	4.1		8
10	S63845/Dactolisib	5.9	3.9	5.8	5.1	3.4		4.7				8.7	7.0		8
11	AZD8055/MK-2206	3.3			3.2	10.2	13.0	14.1				14.3	5.1		7
12	AZD8055/Trametinib	3.7	3.2			14.2	10.5	13.6				12.9	4.2		7
13	Navitoclax/Ibrutinib		3.5			9.9	10.0	8.1				14.4	10.5	3.2	7
14	Navitoclax/Crizotinib		3.7			7.7	9.0	11.5				14.2	8.6	4.8	7
15	Navitoclax/Paclitaxel		3.2			6.5	9.1	7.6				13.3	9.0	4.3	7
16	Navitoclax/LY2784544		3.2			5.9	8.9	7.8				12.6	9.9	4.3	7
17	Navitoclax/Sorafenib		3.1		3.5	3.9	7.4	11.0				12.7	9.3		7
18	AZD8055/Dactolisib			5.7		8.0	8.9	10.1	5.6			9.3	3.2		7
19	AZD8055/Erlotinib	3.3				7.9	9.1	11.3				10.6	4.5	3.1	7
20	S63845/Trametinib	4.0	3.0		3.8	5.6		12.7				12.8	6.0		7
21	AZD8055/pemetrexed		3.3		3.8	8.2	7.2	9.1				10.8	4.4		7
22	S63845/Lapatinib	4.5	3.2			3.6	3.7	5.3				5.6	5.5		7
23	S63845/GDC-0941	8.7				15.2	10.5	12.8				13.1	6.5		6
24	Navitoclax/Osimertinib					15.4	13.6	4.5				13.3	10.1	3.3	6
25	Navitoclax/MK-2206		4.0			10.7	13.5	8.4				15.5	7.5		6

Epithelioid = E
Biphasic = B
Sarcomatoid = S

	**Drug treatments**	**Total E hits (6)**	**Total % hits E**	**Total B hits (6)**	**Total % hits B**
1	Navitoclax/S63845	6	100.0	6	100.0
2	Navitoclax/AZD8055	5	83.3	6	100.0
3	S63845/AZD8055	6	100.0	3	50.0
4	Navitoclax/GDC-0941	4	66.7	4	66.7
5	Navitoclax/Dactolisib	5	83.3	3	50.0
6	Navitoclax	4	66.7	4	66.7
7	Navitoclax/Trametinib	4	66.7	3	50.0
8	AZD8055/GDC-0941	5	83.3	3	50.0
9	Crizotinib/AZD8055	5	83.3	3	50.0
10	S63845/Dactolisib	5	83.3	3	50.0
11	AZD8055/MK-2206	4	66.7	3	50.0
12	AZD8055/Trametinib	4	66.7	3	50.0
13	Navitoclax/Ibrutinib	3	50.0	3	50.0
14	Navitoclax/Crizotinib	3	50.0	3	50.0
15	Navitoclax/Paclitaxel	3	50.0	3	50.0
16	Navitoclax/LY2784544	3	50.0	3	50.0
17	Navitoclax/Sorafenib	4	66.7	3	50.0
18	AZD8055/Dactolisib	3	50.0	4	66.7
19	AZD8055/Erlotinib	3	50.0	3	50.0
20	S63845/Trametinib	4	66.7	3	50.0
21	AZD8055/pemetrexed	4	66.7	3	50.0
22	S63845/Lapatinib	4	66.7	3	50.0
23	S63845/GDC-0941	3	50.0	3	50.0
24	Navitoclax/Osimertinib	2	33.3	3	50.0
25	Navitoclax/MK-2206	3	50.0	3	50.0

Table: S5: Malignant pleural mesothelioma patient oncopanel results

Patient Sample	Tier 1 Variants	Tier 2 Variants	Tier 3 Variants	Tier 4 Variants
MPS:A	None identified.	None identified.	TP53 c.681dupT (p.D228*), exon 7 - in 23% of 312 reads **	MGA c.3322delC (p.P1108Qfs*25), exon 9 - in 64% of 301 reads ***<br>CALR c.92-3_92-2insA (), exon 0 - in 14% of 451 reads ***<br>POLB c.52A>G (p.M18V), exon 1 - in 45% of 281 reads ***<br>TET1 c.998G>T (p.G333V), exon 2 - in 58% of 579 reads ***<br>MDM2 c.851T>G (p.V284G), exon 10 - in 39% of 378 reads ***<br>CREBBP c.2596A>G (p.M866V), exon 14 - in 46% of 279 reads ***
MPS:B	None identified.	None identified.	B2M c.188_189delGA (p.R65Nfs*2), exon 2 - in 33% of 449 reads **<br>SPOP c.172dupT (p.S58Ffs*6), exon 4 - in 30% of 292 reads **<br>DNMT3A c.1919_1920delTT (p.F640*), exon 16 - in 7% of 352 reads **<br>MSH2 c.366delG (p.A123Lfs*51), exon 2 - in 30% of 168 reads **<br>ARID1B c.2515delC (p.P839Lfs*4), exon 8 - in 28% of 548 reads **<br>NF2 c.1612C>T (p.Q538*), exon 15 - in 27% of 249 reads **	POLE c.4150-5G>A, exon 0 - in 9% of 284 reads ***<br>MAP2K2 c.691C>T (p.R231C), exon 6 - in 33% of 207 reads ***
MPS:C	None identified.	None identified.	NF2 c.1156G>T (p.E386*), exon 12 - in 19% of 270 reads **	APC c.8213T>C (p.I2738T), exon 16 - in 36% of 596 reads ***<br>CARD11 c.2889C>A (p.Y963*), exon 22 - in 56% of 460 reads ***<br>FANCI c.233C>T (p.S78L), exon 4 - in 56% of 482 reads ***
MPS:D	None identified.	None identified.	TP53 c.814G>A (p.V272M), exon 8 - in 37% of 276 reads **<br>NF2 c.241-1G>T (), exon 0 - in 21% of 145 reads **	SF3B1 c.2219G>A (p.G740E), exon 15 - in 4% of 252 reads ***<br>WWTR1 c.544A>G (p.M182V), exon 4 - in 48% of 199 reads ***<br>NSD1 c.1686C>G (p.N562K), exon 5 - in 7% of 372 reads ***<br>KAT6B c.3518A>G (p.E1173G), exon 17 - in 57% of 209 reads ***<br>SH2B3 c.10C>A (p.P4T), exon 2 - in 71% of 42 reads ***<br>EME1 c.691A>G (p.R231G), exon 2 - in 55% of 385 reads ***<br>C17orf70 c.1303G>A (p.D435N), exon 4 - in 59% of 259 reads ***<br>TCF3 c.1586+47G>A (), exon 0 - in 62% of 104 reads ***<br>NOTCH3 c.274G>C (p.V92L), exon 3 - in 60% of 275 reads ***<br>ARAF c.1156G>A (p.E386K), exon 11 - in 29% of 351 reads ***
MPS:E	None identified.	None identified.	None identified.	None identified.
MPS:F	None identified.	None identified.	None identified.	None identified.
MPS:G	None identified.	None identified.	SETD2 c.4885C>T (p.H1629Y), exon 7 - in 18% of 226 reads **<br>NF2 c.571T>G (p.W191G), exon 6 - in 4% of 338 reads **	RIF1 c.3452C>T (p.S1151L), exon 30 - in 54% of 273 reads ***
MPS:H	None identified.	None identified.	NF2 c.361delC (p.Q121Rfs*2), exon 3 - in 59% of 334 reads **<br>SETD2 c.3481_3490delTCTCATCCTC (p.S1161Rfs*13), exon 3 - in 39% of 383 reads **<br>BAP1 c.11delG (p.G4Afs*68), exon 1 - in 60% of 96 reads **	DNMT3A c.2724T>G (p.Y908*), exon 23 - in 3% of 548 reads ***<br>XPC c.1546G>A (p.D516N), exon 9 - in 47% of 789 reads ***<br>SETD2 c.3602A>G (p.E1201G), exon 3 - in 59% of 509 reads ***<br>PBRM1 c.2966-43C>T (p.R1000*), exon 19 - in 61% of 261 reads ***<br>CBLB c.1959+1G>A (), exon 0 - in 46% of 450 reads ***<br>ARID1B c.6581G>T (p.R2194L), exon 20 - in 75% of 702 reads ***<br>BRD3 c.662C>T (p.P221L), exon 5 - in 76% of 168 reads ***<br>KAT6B c.4252C>T (p.P1418S), exon 18 - in 44% of 496 reads ***<br>FANCI c.1405T>G (p.Y469D), exon 15 - in 37% of 487 reads ***<br>CBFA2T3 c.1225A>C (p.M409L), exon 9 - in 31% of 156 reads ***
MPS:I	None identified.	None identified.	None identified.	None identified.
MPS:J	None identified.	None identified.	TERT - in 50% of 6 reads **	DIS3 c.1995dupA (p.F666Ifs*15), exon 16 - in 6% of 162 reads ***<br>COL7A1 c.2006G>A (p.R669Q), exon 15 - in 49% of 104 reads ***<br>MECOM c.2289C>A (p.C763*), exon 12 - in 28% of 169 reads ***<br>RECQL4 c.355-8C>T (), exon 0 - in 63% of 60 reads ***<br>KMT2A c.4818G>A (p.M1606I), exon 15 - in 35% of 187 reads ***<br>TSC2 c.721G>A (p.V241I), exon 8 - in 48% of 90 reads ***
MPS:K	None identified.	None identified.	BAP1 c.1993C>T (p.Q665*), exon 16 - in 26% of 339 reads **<br>CDKN2B c.178C>T (p.R60C), exon 2 - in 56% of 79 reads **	MTOR c.4922C>T (p.S1641F), exon 35 - in 47% of 323 reads ***<br>MSH6 c.3557G>A (p.G1186D), exon 7 - in 49% of 252 reads ***<br>FGFR4 c.1711G>A (p.D571N), exon 13 - in 50% of 189 reads ***<br>FLT3 c.2725G>C (p.D909H), exon 22 - in 61% of 291 reads ***<br>BRCA1 c.396C>A (p.N132K), exon 6 - in 53% of 516 reads ***<br>BCOR c.779C>G (p.S260C), exon 4 - in 35% of 356 reads ***
MPS:L & M	None identified.	None identified.	CDKN1A - in 32% of 269 reads **	ERCC2 c.2083C>T (p.R695C), exon 22 - in 47% of 255 reads ***<br>ERCC1 c.152C>T (p.T51I), exon 2 - in 42% of 55 reads ***<br>NF2 c.172G>A (p.E58K), exon 2 - in 11% of 138 reads ***

Table S6: Malignant pleural mesothelioma patient clinical data

MPM ID:	Histology	Clinical stage at time of surgery	Pre-surgery treatment	Post-surgery treatment	Clinical response of patient
MPS:A	Epithelioid	T2 N0 M0	Treatment naïve	4 cycles of adjuvant cisplatin/pemetrexed in 4 months following surgery	Chemotherapy refractory - patient had a partial response but disease progressed/recurrence 6 months following treatment; Survival after surgery = 394 days
MPS:B	Epithelioid	Unkown	Treatment naïve	1 cycle of carboplatin and pemetrexed chemotherapy 1 month following surgery	Chemotherapy resistant - patient had no response to therapy; survival after surgery = 49 days
MPS:C	Biphasic	Unkown	Treatment naïve	6 cycles of carboplatin chemo 2 months after operation to treat metastatic recurrence; 9 cycles pemetrexed maintenance 6 months after surgery	Chemotherapy sensitive - following several rounds of chemotherapy, patient had no evidence of new or growing disease; survival after surgery = 1275+ days
MPS:D	Epithelioid	pT4 N0	Treatment naïve	None	No adjuvant treatment; survival after surgery = 62 days
MPS:E	Biphasic	ypT4 N0	4 cycles of pemetrexed-carboplatin chemotherapy & radiation to chest wall mass completed 2 months prior to surgery	Unkown	Sensitive to chemotherapy applied prior to surgery and to resection - patient has had no evidence of recurrence (notes for this patient stop, unsure of survival status)
MPS:F	Biphasic	Unkown	Previous pleurectomy decortication with installed heat chemotherapy 2 years before, underwent radiation therapy 4 months before, underwent an unkown number of cycles of an unknown chemotherapy	Unkown	Patient had original pleurectomy and had chest wall recurrence - Then had 30 days radiation and then chest ablation; survival after surgery = 524 days
MPS:G	Biphasic	Unkown	neoadjuvant chemotherapy with carboplatin/pemetrexed 2 months prior to surgery	None	No adjuvant therapy; survival after surgery = 151 days
MPS:H	Biphasic	pT2 N0	Treatment naïve	4 cycles of adjuvant chemotherapy (Cisplatin & Pemetrexed) 3 months after surgery, completed; pembrolizumab immunotherapy	Chemotherapy refractory - initially patient responded well but confirmed recurrence 6 months later, started immunotherapy but disease continued to slowly progress; survival after surgery = 899 days
MPS:I	Biphasic	pT2 N1	Treatment naïve	2 cycles of carboplatin 2 months after surgery, 2 cycles of nivolumab immunotherapy 4 months after surgery	Chemotherapy refractory - initially patient responded well but confirmed recurrence, 5 months after surgery (& 2 months after completing chemotherapy), started immunotherapy but disease continued to progress; survival after surgery = 188 days
MPS:J	Sarcomatoid	pT4 N0	Treatment naïve	4 cycles of adjuvant chemo (Cisplatin & Pemetrexed) completed - 6 months after surgery	Chemotherapy refractory - initially patient responded well but confirmed recurrence 11 months after surgery (and 7 months after completing chemotherapy), disease continued to progress; notes for this patient stop, unsure of survival status
MPS:K	Epithelioid	T1b N2 M0	nivolumab about 10 months prior	Pembrolizumab -present	Chemotherapy sensitive - patient has no uptake or abnormality suspicious for disease (no evidence of metastasis); survival after surgery = 735+ days
MPS:L/M	Epithelioid	Unkown	Treatment naïve	Pemetrexed/Cisplatin 9 months after surgery	Chemotherapy resistant - patient had recurrence 9 months after surgery & began chemotherapy, disease continued to progress; survival after surgery = 469 days

Table S7: Most common top hits across all MPM patient samples

		MPS:A	MPS:B	MPS:C	MPS:D	MPS:E	MPS:F	MPS:G	MPS:H	MPS:I	MPS:J	MPS:K	MPS:L	MPS:M	
	Drug treatments	Z-score	Z-score	Z-score	Z-score	Z-score	Z-score	Z-score	Z-score	Z-score	Z-score	Z-score	Z-score	Z-score	Total
1	Navitoclax/S63845	7.9	4.9	13.0	16.0	3.2	4.0	6.5	11.9	12.9	3.8	6.3	25.7	29.9	13
2	Navitoclax/AZD8055	4.0	4.4	14.1	16.1	4.9	3.8	4.6	16.6	10.8			28.9	25.6	11
3	S63845/AZD8055	9.4	3.2	13.2	8.0				15.3	7.8		3.2	22.0	13.1	9
4	Navitoclax/GDC-0941		4.0	10.5				4.8	15.6	10.5	3.7	4.1	15.8	12.9	9
5	Navitoclax/Dactolisib		4.3	7.0	18.5		4.4			9.4	3.6	5.2	7.1	10.5	9
6	Navitoclax		3.3	8.3	16.4		4.2		10.4	8.0	4.2		6.4	7.0	9
7	Navitoclax/Trametinib		4.4	8.3					11.2	12.0	3.8	3.2	12.2	13.8	8
8	AZD8055/GDC-0941	4.3	3.0	13.8					13.0	5.2		3.2	11.5	13.6	8
9	Crizotinib/AZD8055	3.1	3.1	13.3					13.2	4.1		3.5	11.1	7.7	8
10	S63845/Dactolisib	5.9	3.9	4.7	5.8				8.7	7.0		5.1	3.4		8
11	AZD8055/MK-2206	3.3		14.1					14.3	5.1		3.2	10.2	13.0	7
12	AZD8055/Trametinib	3.7	3.2	13.6					12.9	4.2			14.2	10.5	7
13	Navitoclax/Ibrutinib		3.5	8.1					14.4	10.5	3.2		9.9	10.0	7
14	Navitoclax/Crizotinib		3.7	11.5					14.2	8.6	4.8		7.7	9.0	7
15	Navitoclax/Paclitaxel		3.2	7.6					13.3	9.0	4.3		6.5	9.1	7
16	Navitoclax/LY2784544		3.2	7.8					12.6	9.9	4.3		5.9	8.9	7
17	Navitoclax/Sorafenib		3.1	11.0					12.7	9.3		3.5	3.9	7.4	7
18	AZD8055/Dactolisib			10.1	5.7	5.6			9.3	3.2			8.0	8.9	7
19	AZD8055/Erlotinib	3.3		11.3					10.6	4.5	3.1		7.9	9.1	7
20	S63845/Trametinib	4.0	3.0	12.7					12.8	6.0		3.8	5.6		7
21	AZD8055/pemetrexed		3.3	9.1					10.8	4.4		3.8	8.2	7.2	7
22	S63845/Lapatinib	4.5	3.2	5.3					5.6	5.5			3.6	3.7	7
23	S63845/GDC-0941	8.7		12.8					13.1	6.5			15.2	10.5	6
24	Navitoclax/Osimertinib			4.5					13.3	10.1	3.3		15.4	13.6	6
25	Navitoclax/MK-2206		4.0	8.4					15.5	7.5			10.7	13.5	6

Table S8: Highest mean Z-score top hits across all MPM patient samples

		MPS:A	MPS:B	MPS:C	MPS:D	MPS:E	MPS:F	MPS:G	MPS:H	MPS:I	MPS:J	MPS:K	MPS:L	MPS:M	
	Drug treatments	Z-score	Z-score	Z-score	Z-score	Z-score	Z-score	Z-score	Z-score	Z-score	Z-score	Z-score	Z-score	Z-score	Mean
1	Navitoclax/AZD2014								12.4	11.7	1.9	3.7	26.8	23.5	13.3
2	Navitoclax/S63845	7.9	4.9	13.0	16.0	3.2	4.0	6.5	11.9	12.9	3.8	6.3	25.7	29.9	11.2
3	Navitoclax/AZD8055	4.0	4.4	14.1	16.1	4.9	3.8	4.6	16.6	10.8	2.9	1.3	28.9	25.6	10.6
4	S63845/AZD2014								17.4	9.4	1.0	2.5	17.6	13.3	10.2
5	Navitoclax/GDC-0941	2.2	4.0	10.5				4.8	15.6	10.5	3.7	4.1	15.8	12.9	8.4
6	Navitoclax/Trametinib	1.7	4.4	8.3					11.2	12.0	3.8	3.2	12.2	13.8	7.8
7	S63845/AZD8055	9.4	3.2	13.2	8.0	1.9	2.2	-0.7	15.3	7.8	1.5	3.2	22.0	13.1	7.7
8	Navitoclax/Osimertinib	1.2	2.9	4.5					13.3	10.1	3.3	3.0	15.4	13.6	7.5
9	AZD8055/Trametinib	3.7	3.2	13.6					12.9	4.2	2.1	1.9	14.2	10.5	7.4
10	GDC0941/AZD2014								11.7	5.5	-0.9	2.7	12.2	13.0	7.4
11	S63845/GDC-0941	8.7	2.6	12.8				-0.5	13.1	6.5	1.5	2.6	15.2	10.5	7.3
12	AZD8055/MK-2206	3.3	2.4	14.1					14.3	5.1	-0.7	3.2	10.2	13.0	7.2
13	Navitoclax/MK-2206	1.3	4.0	8.4					15.5	7.5	2.7	0.9	10.7	13.5	7.2
14	Navitoclax/Everolimus	1.0		7.4					9.6	11.4	1.7	0.2	13.5	11.6	7.1
15	Navitoclax/Crizotinib	1.4	3.7	11.5					14.2	8.6	4.8	1.9	7.7	9.0	7.0
16	Navitoclax/Ibrutinib	0.5	3.5	8.1					14.4	10.5	3.2	1.9	9.9	10.0	6.9
17	AZD8055/GDC-0941	4.3	3.0	13.8				-1.3	13.0	5.2	1.5	3.2	11.5	13.6	6.8
18	Crizotinib/AZD8055	3.1	3.1	13.3					13.2	4.1	0.1	3.5	11.1	7.7	6.6
19	Navitoclax/Dactolisib	1.7	4.3	7.0	18.5	-0.1	4.4		6.6	9.4	3.6	5.2	7.1	10.5	6.5
20	S63845/MK-2206	5.5	1.8	7.0					14.7	8.7	0.1	2.2	8.8	9.3	6.5
21	Navitoclax/Sorafenib	1.9	3.1	11.0					12.7	9.3	2.9	3.5	3.9	7.4	6.2
22	MK2206/AZD2014								13.5	3.2	-0.3	1.0	7.8	11.7	6.2
23	Navitoclax/LY2784544	1.2	3.2	7.8					12.6	9.9	4.3	1.6	5.9	8.9	6.1
24	Navitoclax/pemetrexed		3.3	4.0				6.2	6.0	10.6	3.1	1.6	9.1	11.0	6.1
25	Osimertinib/AZD8055	3.0	2.0	10.6					11.6	4.2	0.8	3.2	11.6	7.9	6.1

Table S9: Most common top hits across all MPM PDX samples

		0011x 2.1 LF	0011x 2.1 RF	0011x 2.2 LF	0011x 2.2 RF	0184x 3.3 LF	0106x 3.2 RF	
	Drug treatment	Z-score	Z-score	Z-score	Z-score	Z-score	Z-score	Total
1	Navitoclax/S63845	3.7		6.9	4.4	6.0	4.1	5.0
2	AZD8055/Navitoclax	3.3	3.1	7.7	6.1		4.5	5.0
3	GDC-0941/S63845	5.0	3.4	5.3	3.2	5.2		5.0
4	AZD2014/S63845			6.3	3.8	5.9	9.5	4.0
5	AZD2014/Navitoclax		3.6	7.2	3.1		10.1	4.0
6	AZD2014/MK-2206	3.0	3.4	6.2			7.3	4.0
7	MK2206/Navitoclax	4.9	3.7	5.9	4.3			4.0
8	Dactolisib/S63845			4.5	3.0	3.0	4.6	4.0
9	Etoposide/Navitoclax	3.6		5.0	5.5			3.0
10	Ibrutinib/Navitoclax	3.5		5.1	5.2			3.0
11	Palbociclib/Navitoclax			4.6	4.2		4.9	3.0
12	AZD8055/S63845			5.2	3.1	5.1		3.0
13	GDC0941/Navitoclax	3.4		6.3	3.5			3.0
14	MK2206/S63845	3.4		3.7		6.0		3.0
15	Navitoclax			5.4	3.4		3.8	3.0
16	Venetoclax/Navitoclax	3.9		4.8	3.8			3.0
17	Trametinib/S63845		3.3	4.1		4.9		3.0
18	Erlotinib/Navitoclax	4.2		4.6	3.2			3.0
19	AZD2014/Sorafenib	3.1		3.1			5.5	3.0
20	Navitoclax/Osimertinib	4.2		3.1	4.2			3.0
21	Gefitinib/Navitoclax	3.4		3.2	4.8			3.0
22	LY2784544/Navitoclax	3.5		4.0	3.6			3.0
23	Trametinib/AZD8055			4.7		3.1	3.2	3.0
24	Crizotinib/Navitoclax	3.1		3.1	3.4			3.0
25	Dactolisib/MK-2206			6.9			7.2	2.0

Table S10: Highest Mean Z-score top hits across all MPM PDX samples

		0011x 2.1 LF	0011x 2.1RF	0011x 2.2 LF	0011x 2.2 RF	0106x 3.2 RF	0184x 3.3 LF	
		Z-score	Z-score	Z-score	Z-score	Z-score	Z-score	Mean Z-score for MPM PDX's
1	AZD2014/S63845	2.3	2.1	6.3	3.8	9.5	5.9	5.0
2	AZD2014/Navitoclax	2.6	3.6	7.2	3.1	10.1	2.6	4.9
3	Navitoclax/S63845	3.7	2.8	6.9	4.4	4.1	6.0	4.7
4	AZD8055/Navitoclax	3.3	3.1	7.7	6.1	4.5	2.6	4.5
5	AZD2014/MK-2206	3.0	3.4	6.2	2.6	7.3	2.3	4.1
6	MK2206/Navitoclax	4.9	3.7	5.9	4.3	3.1	2.1	4.0
7	GDC0941/S63845	5.0	3.4	5.3	3.2	0.8	5.2	3.8
8	MK2206/S63845	3.4	2.9	3.7	2.3	3.8	6.0	3.7
9	Dactolisib/MK2206	1.5	2.1	6.9	1.0	7.2	2.9	3.6
10	Dactolisib/Navitoclax	2.6	2.2	6.2	2.7	5.6	1.8	3.5
11	AZD8055/MK-2206	3.1	2.5	5.4	3.9	3.3	2.4	3.4
12	AZD2014/GDC-0941	2.0	2.5	6.5	2.2	5.0	1.7	3.3
13	GDC-0941/Navitoclax	3.4	2.3	6.3	3.5	1.8	2.4	3.3
14	Trametinib/S63845	2.5	3.3	4.1	2.6	1.8	4.9	3.2
15	Everolimus/S63845	2.5	1.9	6.4	2.8	1.9	3.6	3.2
16	Dactolisib/S63845	1.2	2.4	4.5	3.0	4.6	3.0	3.1
17	Lapatinib/Navitoclax	3.5	1.6	4.7	2.9	4.9	1.0	3.1
18	Everolimus/Navitoclax	2.8	1.9	5.5	2.5	4.8	0.9	3.1
19	Pemetrexed/Navitoclax	2.2	2.0	5.6	2.6	5.8	0.0	3.1
20	Dactolisib/GDC0941	1.9	2.7	5.2	2.1	5.4	1.1	3.0
21	Sunitinib/Navitoclax	2.7	2.5	2.0	3.3	6.1	1.6	3.0
22	AZD8055/S63845	2.4	2.9	5.2	3.1	-0.7	5.1	3.0
23	AZD2014/Ibrutinib	1.1	0.8	4.1	0.9	8.8	2.2	3.0
24	Palbociclib/Navitoclax	2.7	1.1	4.6	4.2	4.9	0.3	3.0
25	Ibrutinib/Navitoclax	3.5	2.6	5.1	5.2	0.4	0.8	2.9

Table S11: BIM BH3 peptide EC50 in CPDM_0011x MPM PDX model

Treatment	BIM (EC50 ± 95% CI)	Significance*
Vehicle	0.89 (0.84-0.94)	
100 mg/kg Navitoclax	0.54 (0.16-0.92)	0.074
16 mg/kg AZD8055	0.49 (0.42-0.56)	0.00038
Navitoclax + AZD8055	0.49 (0.39-0.59)	0.00094

*One-tailed unpaired t test versus vehicle-treated BIM EC50 for same CPDM_0011x MPM PDX model

Table S12: AZD8055 AUC p-values after Navitoclax or A-1331852 treatment

H2052

Treatment	AZD8055 (AUC ± 95% CI)	Significance*
DMSO	225.1 (206.7-243.4)	
1 μM Navitoclax	185.5 (172.1-198.9)	0.0082
1 μM A-1331852	144.4 (123.9-164.9)	0.0008

*One-tailed unpaired t test versus DMSO-treated AZD8055 AUC for same cell line.

JMN

Treatment	AZD8055 (AUC ± 95% CI)	Significance*
DMSO	206.7 (194.7-218.7)	
1 μM Navitoclax	180.2 (169.7-190.8)	0.0094
1 μM A-1331852	166.8 (157.4-176.2)	0.0015

*One-tailed unpaired t test versus DMSO-treated AZD8055 AUC for same cell line.

JMN1B

Treatment	AZD8055 (AUC 95% CI)	Significance*
DMSO	196.7 (181.2-212.2)	
1 μM Navitoclax	182.9 (178.2-187.6)	0.079
1 μM A-1331852	179.5 (167.3-191.7)	0.067

*One-tailed unpaired t test versus DMSO-treated AZD8055 AUC for same cell line.

MST0-211H

Treatment	AZD8055 (AUC ± 95% CI)	Significance*
DMSO	216.5 (197.6-235.3)	
1 μM Navitoclax	179.6 (178.1-181.1)	0.0067
1 μM A-1331852	164.5 (142.4-186.6)	0.0062

*One-tailed unpaired t test versus DMSO-treated AZD8055 AUC for same cell line.
