# Supplementary material for: Dynamic BH3 profiling identifies pro-apoptotic drug combinations for the treatment of malignant pleural mesothelioma
Source: Nat Commun. 2023 May 20;14:2897. doi: 10.1038/s41467-023-38552-z (PMC10199949; doi:10.1038/s41467-023-38552-z)
Supplement: Supplementary file 2 — Reporting Summary [file 41467_2023_38552_MOESM2_ESM.pdf]

nature portfolio

Corresponding author(s): Anthony Letai

Last updated by author(s): Mar 27, 2023

Reporting Summary

Nature Portfolio wishes to improve the reproducibility of the work that we publish. This form provides structure for consistency and transparency in reporting. For further information on Nature Portfolio policies, see our Editorial Policies and the Editorial Policy Checklist.

Statistics

For all statistical analyses, confirm that the following items are present in the figure legend, table legend, main text, or Methods section.

n/a	Confirmed	
☐	☒	The exact sample size (*n*) for each experimental group/condition, given as a discrete number and unit of measurement
☐	☒	A statement on whether measurements were taken from distinct samples or whether the same sample was measured repeatedly
☐	☒	The statistical test(s) used AND whether they are one- or two-sided<br>*Only common tests should be described solely by name; describe more complex techniques in the Methods section.*
☐	☒	A description of all covariates tested
☐	☒	A description of any assumptions or corrections, such as tests of normality and adjustment for multiple comparisons
☐	☒	A full description of the statistical parameters including central tendency (e.g. means) or other basic estimates (e.g. regression coefficient) AND variation (e.g. standard deviation) or associated estimates of uncertainty (e.g. confidence intervals)
☐	☒	For null hypothesis testing, the test statistic (e.g. *F*, *t*, *r*) with confidence intervals, effect sizes, degrees of freedom and *P* value noted<br>*Give P values as exact values whenever suitable.*
☐	☒	For Bayesian analysis, information on the choice of priors and Markov chain Monte Carlo settings
☐	☒	For hierarchical and complex designs, identification of the appropriate level for tests and full reporting of outcomes
☐	☒	Estimates of effect sizes (e.g. Cohen's *d*, Pearson's *r*), indicating how they were calculated

Our web collection on statistics for biologists contains articles on many of the points above.

Software and code

Policy information about availability of computer code

Data collection	BD Fortessa and ImageXpress Micro Confocal High-Content Microscope.
Data analysis	Graphpad Prism 8, Microsoft excel, Image J colony area plugin, BD FACSDiva software and MetaXpress.

For manuscripts utilizing custom algorithms or software that are central to the research but not yet described in published literature, software must be made available to editors and reviewers. We strongly encourage code deposition in a community repository (e.g. GitHub). See the Nature Portfolio guidelines for submitting code & software for further information.

Data

Policy information about availability of data

All manuscripts must include a data availability statement. This statement should provide the following information, where applicable:

- Accession codes, unique identifiers, or web links for publicly available datasets
- A description of any restrictions on data availability
- For clinical datasets or third party data, please ensure that the statement adheres to our policy

All data needed to evaluate the conclusions in the paper are present in the article, source data and supplementary files.

Field-specific reporting

Life sciences study design

All studies must disclose on these points even when the disclosure is negative.

Sample size	No sample size for in vitro experiments was determine, so we used n=3 independent experiments. Therefore, all in vitro cell line experiments were done at least in 3 biological replicates and each independent experiment, where possible, was also carried out using technical repeats. MPM patient samples were precious with only one sample per patient because you receive the sample after the patient has surgery for tumor resection. Due to this limited patient sample size we did all these experiments in technical duplicates. We wanted to treat the MPM PDX tumor samples the same as we did for the patient so for each tumor harvested we carried out CROCS HTDBP in technical duplicates. We implanted MPM PDX tumors in the right and left flank of two mice, so each model had the chance to grow up to 4 tumors per model. However, not all sites grew tumors. We did not use a statistical method to decide sample size for animal studies but keeping the 3R's in mind we did not want to use too few mice and jeopardize reliability of the results or use too many mice, because the risk making a false positive conclusion increases. With that in mind and with our previous animal study experience we decided to use 7-9 mice per arm for the in vivo efficacy study. Three mice for the pharmacodynamic study (iBH3 profiling and Western blotting analysis).
Data exclusions	All animals that were implanted with CPDM_0011x tumors were enrolled in the efficacy study or pharmacodynamic study (iBH3 profiling and Western blotting analysis). Because the tumor take rate was 100% for this model, no animals were excluded from this study. The take rate for CPDM_0106x tumor growth and CPDM_0184x tumor growth was 50%. There were no exclusion criteria for in vitro cell line data or MPM patient sample ex vivo data. All data was included in the manuscript.
Replication	All in vitro experiments were carried out in 3 biological replicates. All replicates were successful and we included all replicates of experiments in our data analysis. CROCS HTDBP was carried out in technical duplicates because patient samples are precious.
Randomization	For in vivo efficacy studies mice bearing CPDM_0011x tumors (Figure 4a-d), measuring between 150-250 mm3 were randomized, using the deterministic method, into 5 groups of 7-9 mice in a rolling recruitment method. This method was also used for in vivo pharmacodynamic studies when tumors reached ~ 800 mm3 (Figure 4e-f) . No randomization method was needed for data generated else where in the paper because we used all available patient tumors and MPM PDX tumors for ex vivo CROCS HT-DBP screen.
Blinding	We were blinded to any identifying information from patients who signed up for the study.

Reporting for specific materials, systems and methods

We require information from authors about some types of materials, experimental systems and methods used in many studies. Here, indicate whether each material, system or method listed is relevant to your study. If you are not sure if a list item applies to your research, read the appropriate section before selecting a response.

Materials & experimental systems

n/a	Involved in the study	
☐	☒	Antibodies
☐	☒	Eukaryotic cell lines
☒	☐	Palaeontology and archaeology
☐	☒	Animals and other organisms
☐	☒	Human research participants
☒	☐	Clinical data
☒	☐	Dual use research of concern

Methods

n/a	Involved in the study	
☒	☐	ChIP-seq
☐	☒	Flow cytometry
☒	☐	MRI-based neuroimaging

Antibodies

Antibodies used	For Western blotting, β-Actin (#4967S 1:2000), AKT (#4685S 1:1000), phosphoSer473-AKT (#4685S 1:1000), BIM (#2933S 1:1000), BAK (#12105S 1;1000), BAX (#2772S 1;1000), BCL-xL (#2764S 1;1000), BCL-2 (#15071S 1;1000), BAK (#12105S 1;1000), cleaved Caspase-3 (#9661S 1:1000), MCL-1 (#39224S 1;1000), PUMA (#4976S 1;1000), PARP (#9542S 1;1000), S6 (#2317S 1;1000) and phosphoSer235/236-S6 (#4857S 1;1000) ((Cell Signaling Technology).<br>For immunoprecipitation, the following antibodies: anti-MCL1 (12 µg; BD Pharmingen), anti-BCL-xL (8 µg; EMD Millipore), and anti-mouse IgG1 (MCL-1 antibody isotype, 12 µg; #5415S) and IgG3 (Bcl-xL antibody isotype, 8 µg; #37988S) isotype controls (Cell Signaling Technology).<br>For DBP and HTDBP, nuclei were stained with Hoechst33342 (1:2000; Life Technologies) to determine total number of cells. A pan cytokeratin antibody (#628608; 1:1000; Biolegend) was used to identify epithelioid MPM tumor cells parent population and vimentin (#677809; 1:1000; Biolegend) was used to identify sarcomatoid MPM parent population. Cytochrome c positive cells % was measured using cytochrome c-Alexa647 antibody (#612310; 1:2000; Biolegend).
Validation	Antibodies for microscopy or flow cytometry (DBP) were validated with positive and negative controls for targets. (or DBP and HTDBP, nuclei were stained with Hoechst33342 (1:2000; Life Technologies) to determine total number of cells. A pan cytokeratin antibody (#628608; 1:1000; Biolegend) was used to identify epithelioid MPM tumor cells parent population and vimentin (#677809; 1:1000; Biolegend) was used to identify sarcomatoid MPM parent population. Cytochrome c positive cells % was measured using cytochrome c-Alexa647 antibody (#612310; 1:2000; Biolegend)).

Antibodies for immunoprecipitation were validated using IgG controls for specific antibody isotypes (anti-MCL1 (12 µg; BD Pharmingen), anti-BCL-xL (8 µg; EMD Millipore), and anti-mouse IgG1 (MCL-1 antibody isotype, 12 µg; #5415S) and IgG3 (Bcl-xL antibody isotype, 8 µg; #37988S) isotype controls (Cell Signaling Technology).

Antibodies for Western blotting analysis were validated using positive control cell line lysate for the target (based on experience and literature, also use BenchSci antibody data base to find the best antibodies for each target). β-Actin (#4967S 1:2000), AKT (#4685S 1:1000), phosphoSer473-AKT (#4685S 1:1000), BIM (#2933S 1:1000), BAK (#12105S 1;1000), BAX (#2772S 1;1000), BCL-xL (#2764S 1;1000), BCL-2 (#15071S 1;1000), BAK (#12105S 1;1000), cleaved Caspase-3 (#9661S 1:1000), MCL-1 (#39224S 1;1000), PUMA (#4976S 1;1000), PARP (#9542S 1;1000), S6 (#2317S 1;1000) and phosphoSer235/236-S6 (#4857S 1;1000) ((Cell Signaling Technology).

Eukaryotic cell lines

Policy information about cell lines

Cell line source(s)	H2052 and MSTO-211H (ATCC). JMN and JMN1B from the Brigham and Women's core (gift from Dr. Bueno at Brigham and Women's Hospital)
Authentication	Cell lines were authenticated using the Promega GenePrint® 10 System (Promega Corporation, WI, USA; authenticated at Dana-Farber Cancer Institute (DFCI), Molecular Biology Core Facility). JMN and JMN1B STR profiles are not available for reference.
Mycoplasma contamination	All cell lines were routinely tested for mycoplasma and were negative.
Commonly misidentified lines (See ICLAC register)	None of the 4 cell lines we used in this manuscript are on the misidentified ICLAC register.

Animals and other organisms

Policy information about studies involving animals; ARRIVE guidelines recommended for reporting animal research

Laboratory animals	Female 8 week old SCID-beige mice (C.B-17/IcrHsd-PrkdcscidLystbg-J; from Envigo) and Female 8 week old NSG (NOD.Cg-Prkdcscid Il2rgtm1Wjl/SzJ, from The Jackson Laboratory #005557).
Wild animals	Study didn't involve wild animals.
Field-collected samples	Study didn't involve samples collected in the field
Ethics oversight	All mice were maintained within the DCFI animal facility and all experiments involving animals were conducted in accordance with the DFCI policy and animal protocol, reviewed and approved by the DFCI Institutional Animal Care and Use Committee.

Note that full information on the approval of the study protocol must also be provided in the manuscript.

Human research participants

Policy information about studies involving human research participants

Population characteristics	Consecutive patients with malignant pleural mesothelioma receiving surgical resection. We do not know any identifying information of the patients such as age, race or gender.
Recruitment	Patients were recruited by Dr. Bueno based on patient undergoing surgical resection for malignant pleural mesothelioma. No other information was made available when receiving patient samples used in the manuscript. We have limited clinical information on patients but what we do have in found in Table S6.
Ethics oversight	Fresh primary malignant pleural mesothelioma tumors obtained from tumor resections, after patients signed an informed consent approved by the Institutional Review Board (#98-063) at Brigham and Women's Hospital and Dana-Farber Cancer Institute.

Note that full information on the approval of the study protocol must also be provided in the manuscript.

Flow Cytometry

Plots

Confirm that:

- [x] The axis labels state the marker and fluorochrome used (e.g. CD4-FITC).
- [x] The axis scales are clearly visible. Include numbers along axes only for bottom left plot of group (a 'group' is an analysis of identical markers).
- [x] All plots are contour plots with outliers or pseudocolor plots.
- [x] A numerical value for number of cells or percentage (with statistics) is provided.

Methodology

Sample preparation	Tumors were dissociated, permeabilized and intracellular BH3 profiling was carried out. Then cells were fixed and stained for flow cytometry.
Instrument	BD Fortessa analyzer
Software	DIVA software (BD Biosciences)
Cell population abundance	10,000 events, approximately 9,000 cells and from that approximately 35-55% mesothelioma cells.
Gating strategy	SSC-A Vs. FSC-A (all cells), SSC-W Vs. SSC-H (singlets), Hoechst Vs. SSC-A (live cells), Pan-cytokeratin-488 Vs. SCC-A (epithelioid parent population), cytochrome c Vs. SSC-A (cytochrome c +ve cells). Gating strategy can be found in the source data file.

[x] Tick this box to confirm that a figure exemplifying the gating strategy is provided in the Supplementary Information.
