# Supplementary material for: Dynamic BH3 profiling identifies pro-apoptotic drug combinations for the treatment of malignant pleural mesothelioma
Source: Nat Commun. 2023 May 20;14:2897. doi: 10.1038/s41467-023-38552-z (PMC10199949; doi:10.1038/s41467-023-38552-z)
Supplement: Supplementary file 3 — Description of Additional Supplementary Files [file 41467_2023_38552_MOESM3_ESM.pdf]

Title: Supplementary Data 1:

Description: Clinically relevant oncology combination screen (CROCS) on primary MPM patient samples using HTDBP to identify hits. Fresh primary MPM patient samples were dissociated, treated with CROCS and HTDBP carried out. Cells were analyzed by immunofluorescence microscopy. Delta priming and Z-score was calculated to identify drug/drug combinations that prime tumor cells. Table show mean Z-score for each drug treatment (carried out in duplicate), for each primary MPM patient sample (MPS). Yellow highlighted drug/s represents a hit drug/s treatment that prime the primary MPM patient cells, with a Z-score ≥ 3 with no replicate <1.5 . Green highlighted drug/s represents a non hit which has Z-score ≥ 3 with one replicate <1.5. None highlighted drug/s are none hits.

Title: Supplementary Data 2:

Description: Clinically relevant oncology combination screen (CROCS) on MPM PDX tumors using HTDBP to identify hits. Fresh MPM PDX tumors were dissociated, treated with CROCS and HTDBP carried out. Cells were analyzed by immunofluorescence microscopy. Delta priming and Z-score was calculated to identify drug/drug combinations that prime tumor cells. Table show mean Z-score for each drug treatment (carried out in duplicate), for each MPM PDX tumor. Yellow highlighted drug/s represents a hit drug/s treatment that prime MPM PDX tumor cells, with a Z-score ≥ 3 with no replicate <1.5 . Green highlighted drug/s represents a non hit which has Z-score ≥ 3 with one replicate <1.5. None highlighted drug/s are none hits.

Title: Supplementary Data 3:

Description: Three Tables showing the hits that overlap between the CROCS HTDBP hits for primary MPM patient tumor samples and MPM PDX tumor samples (Table 1), hits only found in MPM patient samples and not PDX (Table 2) and the hits only found in MPM PDX samples and not patients (Table 3).
